# CXCL4 assembles DNA into liquid crystalline complexes to amplify TLR9-mediated interferon-α production in systemic sclerosis

**DOI:** 10.1038/s41467-019-09683-z

**Published:** 2019-05-01

**Authors:** Roberto Lande, Ernest Y. Lee, Raffaella Palazzo, Barbara Marinari, Immacolata Pietraforte, Giancarlo Santiago Santos, Yves Mattenberger, Francesca Spadaro, Katia Stefanantoni, Nicoletta Iannace, Aleksandra Maria Dufour, Mario Falchi, Manuela Bianco, Elisabetta Botti, Luca Bianchi, Montserrat Alvarez, Valeria Riccieri, Marie-Elise Truchetet, Gerard C.L. Wong, Carlo Chizzolini, Loredana Frasca

**Affiliations:** 1 National Center for Drug Research and Evaluation, Pharmacological research and experimental therapy UNIT, Istituto Superiore di Sanità (ISS), 00161 Rome, Italy; 2 Department of Bioengineering, Department of Chemistry & Biochemistry, and California NanoSystems Institute, University of California, Los Angeles, CA 90095 USA; 3 Dermatology Unit, Department of Systems Medicine, University of Tor Vergata, Rome, 00133 Italy; 4 Department of Oncology and Molecular Medicine, Istituto Superiore di Sanità, 00161 Rome, Italy; 5 Department of Microbiol and Molecular Medicine, University of Geneva, CH-1211 Geneva, Switzerland; 6 Istituto Superiore di Sanità, Confocal Microscopy Unit, Core Facilities, Rome, 00161 Italy; 7 Division of Rheumatology, Internal Medicine and Medical Specialties, University La Sapienza, 00161 Rome, Italy; 8 Immunology & Allergy and Immunology & Pathology, University Hospital and School of Medicine, CH-1211 Geneva, Switzerland; 9 Istituto Superiore di Sanità, National AIDS Center, Rome, 00161 Italy; 10 Division of Rheumatology and immunoConcept, University Hospital, Bordeaux, 33076 France

**Keywords:** Autoimmunity, Chemokines, Toll-like receptors, Systemic sclerosis

## Abstract

Systemic sclerosis (SSc) is a chronic autoimmune disease characterized by fibrosis and vasculopathy. CXCL4 represents an early serum biomarker of severe SSc and likely contributes to inflammation via chemokine signaling pathways, but the exact role of CXCL4 in SSc pathogenesis is unclear. Here, we elucidate an unanticipated mechanism for CXCL4-mediated immune amplification in SSc, in which CXCL4 organizes “self” and microbial DNA into liquid crystalline immune complexes that amplify TLR9-mediated plasmacytoid dendritic cell (pDC)-hyperactivation and interferon-α production. Surprisingly, this activity does not require CXCR3, the CXCL4 receptor. Importantly, we find that CXCL4-DNA complexes are present in vivo and correlate with type I interferon (IFN-I) in SSc blood, and that CXCL4-positive skin pDCs coexpress IFN-I-related genes. Thus, we establish a direct link between CXCL4 overexpression and the IFN-I-gene signature in SSc and outline a paradigm in which chemokines can drastically modulate innate immune receptors without being direct agonists.

## Abstract (web summary)

CXCL4 is an inflammatory chemokine signaling through CXCR3 receptor. Here the authors show a CXCR3-independent function of CXCL4: it forms liquid crystals with DNA, potentiating mammalian and bacterial DNA recognition by TLR9, thereby amplifying interferon-a production in systemic sclerosis.

## Introduction

Systemic sclerosis (SSc) is a complex, heterogeneous autoimmune disorder with widespread fibroproliferative vasculopathy, inappropriate inflammation, and tissue and organ fibrosis^1^. Fibrosis distinguishes SSc from other systemic autoimmune diseases and has a major impact on quality of life and patient survival^1,2^. Although a detailed understanding of the molecular mechanisms driving fibrosis in SSc remains elusive, recent studies imply that dysregulation of the innate immune system in genetically predisposed individuals plays a role. Accumulating evidence strongly suggests that aberrant Toll-like receptor (TLR) activation is central to SSc pathogenesis^3,4^.

Indeed, chronic stimulation of TLR3 and TLR4 maintains a pro-fibrotic SSc-fibroblast phenotype^3–6^. Moreover, TLR activation contributes to production of type-I interferon (IFN-I) and pro-inflammatory cytokines by innate immune cells and fibroblasts^3–6^. Interestingly, an IFN-I-induced gene signature is present in approximately half of the SSc patients and genome-wide association studies identified polymorphisms in IFN-I-related genes^7–11^. Studies suggest that IFN-I blockade could be beneficial in early SSc, where the IFN-I-gene signature is associated with disease severity^8,9,12^. Consistent with this, IFN-I-based therapies induced or aggravated SSc^13^.

A recent proteomic study identified chemokine (C–X–C motif) ligand 4 (CXCL4) (also known as platelet factor 4, PF4) as a biomarker of SSc, particularly in early active diffuse SSc^3^. CXCL4 was detected in SSc skin where it co-localized with plasmacytoid dendritic cells (pDCs)^3^. Moreover, CXCL4-releasing SSc pDCs were shown to overproduce IFN-α when stimulated with synthetic oligonucleotide CpG^3,4^. CXCL4 cutaneous injection amplifies IFN-I-induced genes^3^. Presumably, CXCL4 receptor-specific signaling plays a specific role in inflammation. At present, it is unknown whether there is a mechanistic relationship between CXCL4 expression and IFN-α at the molecular level, and why IFN-α levels are so high.

Here, we show that CXCL4 enables innate immune recognition of natural DNA. Detailed analyses of SSc skin biopsies showed that CXCL4 spatially co-localizes with pDCs and IFN-I-related gene expression, that circulating CXCL4 and IFN-α levels correlate, and that CXCL4–DNA complexes are present in vivo. By analyzing CXCL4–DNA interactions using synchrotron small-angle X-ray scattering (SAXS), we demonstrated that CXCL4 forms liquid crystalline complexes with both human DNA (self-DNA, huDNA) and microbial DNA (bacterial DNA, bacDNA). CXCL4 chaperones and organizes DNA into periodic liquid crystalline structures with an inter-DNA spacing well matched with the steric size of TLR9, enabling strong activation of TLR9 in pDCs. Taken together, these data identify a direct mechanistic link between CXCL4 overexpression and the IFN-I signature in SSc, consistent with a general conceptual framework in which immune activation is modulated by the supramolecular organization of CXCL4–DNA complexes. These findings illuminate unexpected non-agonist functions of CXCL4 in normal immune responses and in diseases characterized by local or systemic hyperexpression of CXCL4, including autoimmune diseases, chronic infections, wound healing, traumas, and cancer^14^.

## Results

### CXCL4 overexpression correlates with IFN-Ι upregulation

**Fig. 1:**
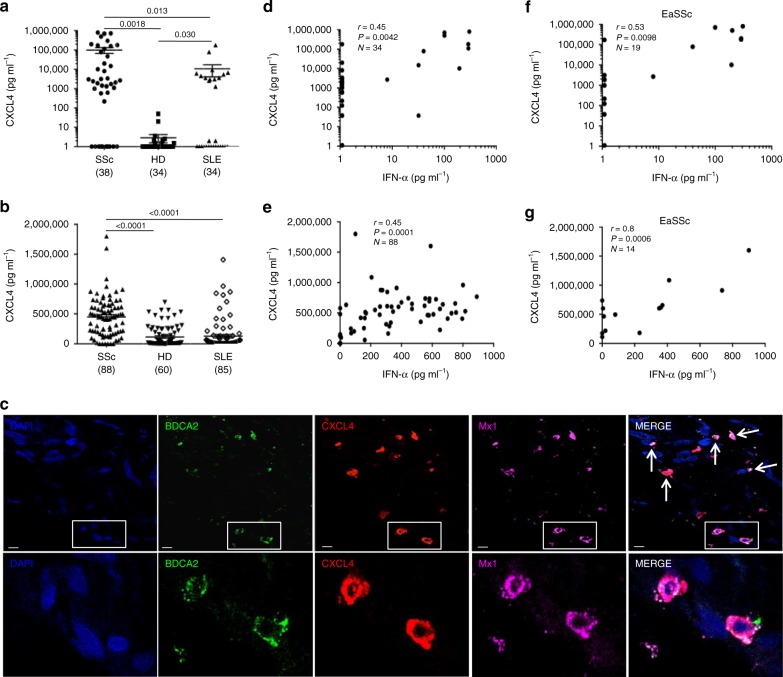
CXCL4 is overexpressed in SSc and correlates with IFN-I. CXCL4 was measured by ELISA in the plasma of SSc (*N* = 38) (discovery cohort), HD (*N* = 34), and SLE (*N* = 34) (**a**), and in the sera of SSc patients (*N* = 88), HD (*N* = 60), and SLE patients (*N* = 85) (**b**). Horizontal bars are the means and vertical bars are the standard errors of the mean (s.e.m.). *P*-values are calculated with Student’s *t* test for unpaired samples (two-tailed). **c** Confocal images of SSc skin stained with DAPI (blue) to color nuclei, anti-BDCA2 (green), anti-Mx1 (magenta), and anti-CXCL4 (red). White arrows indicate co-localization of BDCA2, CXCL4, and Mx1. Upper images show a dermal compartment (objective ×60; bar, 10 µm). Lower images show a detail (inset) of the dermal compartment. One representative experiment of 10 performed with different SSc donors. Amounts of CXCL4 measured in SSc plasma (**d**, **f**) or serum (**e**, **g**) were correlated to IFN-α level measured by ELISA in the same sera/plasma. Correlation was measured by Pearson’s correlations test. Coefficient of correlation “*r*”, significance “*P”*, and sample size “*N*” are indicated

CXCL4 was proposed as biomarker of severe SSc disease^3,4^. We quantitatively assessed CXCL4 levels in the plasma of a discovery (*N* = 38) and in the sera of a replication (*N* = 88) SSc cohort, respectively (Supplementary Table 1) by enzyme-linked immunosorbent assay (ELISA). As controls, we assessed plasma/serum CXCL4 levels in HD and in systemic lupus erythematosus (SLE, Supplementary Table 1). Circulating CXCL4 was significantly more abundant in SSc [mean ± standard deviation (SD): 99,175 ± 209,215 pg ml^−1^, range: 0–805,000 pg ml^−1^, *N* = 38] compared with HD [2.9 ± 8.2 pg ml^−1^, range: 0–50 pg ml^−1^, *N* = 34, *P* = 0.0018] and SLE plasma [10,677 ± 35,742 pg ml^−1^, range: 0–180,000 pg ml^−1^, *N* = 34, *P* = 0.013] (Fig. 1a). The results were comparable in the independent SSc replication cohort (see Suppl. Table 1, Fig. 1b), in which we analyzed sera, revealing again the highest expression of CXCL4 in SSc (449,563 ± 332,000 pg ml^−1^, range: 0–1.8x10^6^ pg ml^–1^, *N* = 88), as compared with HD (114,354 ± 171,987 pg ml^−1^, range: 0–696,050 pg ml^−1^, *N* = 60, *P* < 0.0001) and SLE (127,940 ± 259,133 pg ml^−1^, range: 0–1.4x10^6^ pg ml^−1^, *N* = 85, *P* < 0.0001) (Fig. 1b). Of note, a subgroup of SLE patients showed increased levels of circulating CXCL4 (Fig. 1a, b). Early active SSc (eaSSc, disease duration less than 4 years) either limited/diffuse (*N* = 19, Supplementary Fig. 1a, and *N* = 14, Supplementary Fig. 1b), had the highest absolute level of CXCL4, as compared with long-lasting diffuse SSc (dSSc) (*P* = 0.011; *N* = 21 and *P* = 0.0018, *N* = 38) or limited SSc (lSSc) (*N* = 36, *P* = 0.0013) (Supplementary Fig. 1a, b).

By using confocal laser scanning microscopy (LSM), we found that 20 of 23 SSc skin biopsies analyzed (86.9%) expressed CXCL4 in the dermis, whereas all HD skin biopsies (*N* = 11) were virtually negative for CXCL4 expression (Supplementary Fig. 1c). Infiltrating pDCs were detectable in six of nine (66%) SSc skin biopsies where CXCL4 co-localized with BDCA2, the pDC marker^15–17^ (Supplementary Fig. 1d), although CXCL4-positive cells, which were not pDCs, were also detected. Since pDCs are potent sources of IFN-I, we wondered whether CXCL4 co-localized with IFN-I-induced proteins in pDC. By co-staining SSc skin for CXCL4, Mx1 (an IFN-I-inducible protein^18^) and BDCA2, we found dermal pDCs simultaneously positive for CXCL4 and Mx1 (Fig. 1c).

Further, we tested whether CXCL4 correlated with plasma/serum IFN-I levels in SSc. Ten out of 34 (29%) SSc plasma and 48 out of 88 (54%) SSc sera had detectable IFN-α (Supplementary Fig. 2a, b), which was in statistically significant correlation with CXCL4 in both SSc cohorts (*r* = 0.45, *P* = 0.0042, *N* = 34 and *r* = 0.45, *P* = 0.0001, *N* = 88) (Fig. 1d, e). The strongest correlation was observed in eaSSc (*r* = 0.53, *P* = 0.0098, *N* = 19, discovery cohort, and *r* = 0.8, *P* = 0.0006, *N* = 14, replication cohort; Fig. 1f, g). IFN-α in plasma/sera correlated with CXCL4 both in dSSc (*r* = 0.38, *P* < 0.0001, *N* = 14, discovery cohort, *r* = 0.52, *P* = 0.0007, *N* = 38, replication cohort) and lSSc (*r* = 0.39, *P* = 0.020, *N* = 36, replication cohort). No significant correlation was observed between S100A8/A9 and IFN-α (*r* = −0.15, *P* = 0.11, *N* = 64), or LL37 and IFN-α (*r* = −0.008, *P* = 0.48, *N* = 36) (Supplementary Fig. 3). We assessed the levels of S100A8/A9 (526,600 ± 727,038 pg ml^−1^, range: 0–3.2 × 10^6^ pg ml^−1^ in SSc, 42.6 ± 117.5 pg ml^−1^, range: 0–409.6 pg m^−1^ in HD) and LL37 (1507 ± 1737 pg ml^−1^, range: 0–8437 pg ml^−1^ in SSc, 866.5 ± 666.5 pg ml^−1^, range: 0–2022 pg ml^−1^ in HD), as in Supplementary Fig. 3a, c, because both are potential biomarkers in SSc^19–21^. In contrast, although 14 out of 34 (41%) SLE plasma and seven out of 83 (8%) SLE sera showed increased CXCL4 levels, we did not find a significant correlation between plasma/serum CXCL4 and IFN-α in these patients (*r* = 0.05, *P* = 0.40, *N* = 26, for SLE plasma, *r* = 0.04, *P* = 0.39, *N* = 53, for SLE sera).

These results show that circulating CXCL4 and IFN-α levels positively correlate in a statistically significant manner in SSc and support the hypothesis that CXCL4 plays an important role in the upregulation of IFN-I in SSc.

### CXCL4 complexes and protects natural DNA from degradation

**Fig. 2:**
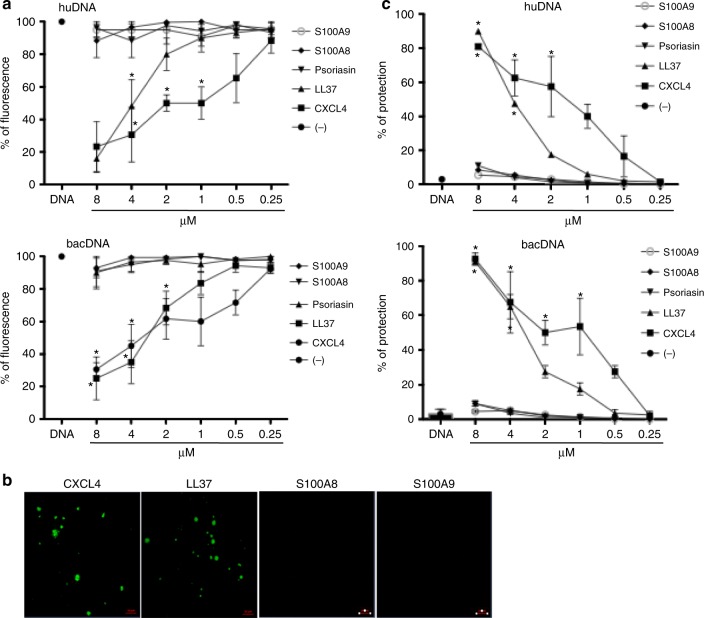
CXCL4 forms complexes with different DNA types and protects DNA from nucleases. **a** HuDNA or bacDNA (10 μg ml^−1^) were stained with PicoGreen and mixed with different concentrations of the indicated molecules. Fluorescence emission was measured by a fluorimeter. Data are expressed as percent of fluorescence with respect to the fluorescence of DNA alone (100%). Horizontal bars represent the mean, vertical bars are s.e.m. Results from eight independent experiments performed with either huDNA or bacDNA (four each); *P-values < 0.05 by Student’s *t* test for paired samples (two-tailed) are calculated with respect to the fluorescence of DNA alone; **b** 10 μM of the indicated proteins were premixed with 10 μg of fluorochrome-conjugated huDNA. Formation of complexes was visualized by confocal microscopy; no binding resulted in a dark panel. One representative experiment out of four. **c** HuDNA or bacDNA (10 μg ml^−1^) were mixed with different doses of the indicated proteins for 20 min in the presence/absence of DNase I (see Methods). Fluorescence was analyzed by PicoGreen assay and percent of DNA protected from degradation calculated with respect to DNA degradation (decrease of picoGreen fluorescence) obtained in the absence of any molecule (DNA alone). Horizontal bars are the mean, vertical bars are s.e.m. Results from six independent experiments performed with huDNA or bacDNA (three each). *P-values < 0.05 by Student’s *t* test for paired samples (two-tailed) calculated in comparison with degradation of DNA alone

CXCL4 contains strongly clustered cationic charges (net charge +3.07 at pH 7.4) and an amphipathic α-helical structure at its COOH terminus, reminiscent of the common core structures in helical antimicrobial peptides (AMPs) such as cathelicidin LL37^14,22,23^. A number of AMPs are known to bind DNA and signal to the immune system through TLRs, including LL37, human β-defensins, and lysozyme^22–25^. CXCL4 is known to bind heparin and other anionic polyelectrolytes^14^. By using a fluorescent dye-stained DNA (PicoGreen)^23,25^, we found that CXCL4 formed complexes with bacDNA (from *E.* *coli*, see Methods) and huDNA (self-DNA) to a similar extent (Fig. 2a). We observed decreasing fluorescence with increasing amounts of CXCL4, suggestive of increasing formation of protein–DNA complexes. LL37–DNA complexes were used as a positive control^22^. No complexes were observed with psoriasin^24,25^ (S100A7), or with either S100A8 or S100A9. We further quantified the ability of CXCL4 to bind DNA, by electrophoretic mobility shift assays (EMSA) using increasing concentrations of CXCL4 and control peptides complexed with plasmid pDB29 DNA (see Methods). CXCL4 began to retard DNA migration at 0.8–1.6 μM, whereas LL37 required a slightly higher concentration (3.2 μM) (Supplementary Fig. 4a). The same experiments were performed by complexing CXCL4 or control LL37 with huDNA. Remarkably, CXCL4 retarded huDNA migration at concentrations between 0.1 and 0.5 μM (Supplementary Fig. 4b). The formation of CXCL4–DNA complexes was also directly visualized using LSM and fluorescent-labeled huDNA (Fig. 2b)^25^. Particulate complexes were observed with CXCL4–huDNA, and with the positive control LL37, but not with S100A8 and S100A9 proteins. Interestingly, CXCL4, S100A8, and S100A9 are all upregulated in SSc^2,3,20^, but only CXCL4 bound appreciably to huDNA/bacDNA in our experiments.

DNA released from cells during inflammation is rapidly degraded by exonucleases/endonucleases. To assess the impact of CXCL4 on such degradation, we incubated plasmid DNA pDB29 with the restriction enzyme EcoRV (endonuclease, see Methods) in the presence/absence of CXCL4. The resulting cleavage products were visualized using gel electrophoresis (Supplementary Fig. 4c). Normal cleavage of pDB29 by EcoRV results in linearization, while lack of cleavage results in relaxed–circular and supercoiled–circular forms. CXCL4 (13% of plasmid was linearized) and to some extent LL37 (88% of plasmid was linearized), but not psoriasin (all used at equimolar concentrations), protected the plasmid from EcoRV digestion. We also incubated CXCL4–huDNA/CXCL4–bacDNA complexes in the presence of DNase I, and fluorescence was quantified using PicoGreen^23,25^. CXCL4 and LL37, but not S100A8 or S100A9 or psoriasin^21^ protected huDNA/bacDNA from degradation by DNase I (Fig. 2c). Overall, these data demonstrate that CXCL4 binds to and protects DNA from different sources from enzymatic degradation.

### PDC activation by CXCL4–DNA complexes depends on DNA size

**Fig. 3:**
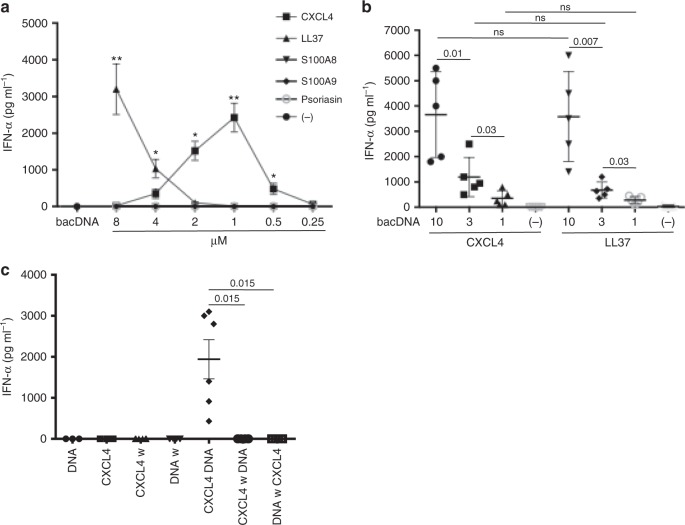
CXCL4–DNA complexes activate pDCs to produce IFN-α. **a** pDCs were stimulated with the indicated doses of CXCL4 or control molecules premixed with bacDNA (10 μg ml^−1^). IFN-α was measured after 24 h by ELISA. Data are shown as a mean±s.e.m. from five independent experiments. *P-values < 0.05, **P-values < 0.01 by Student’s *t* test for paired samples (two-tailed) are calculated with respect to IFN-α values obtained after stimulation of pDCs by bacDNA alone. **b** PDCs were stimulated with complexes made with fixed concentrations of CXCL4 (1 μM) or LL37 (10 μM) and different doses of bacDNA (μg ml^−1^). IFN-α was measured after 24 h by ELISA. Horizontal bars are the means, vertical bars are the s.e.m., *P*-values by Student’s *t* test for paired samples (two-tailed). **c** PDCs were stimulated with CXCL4 (1 μM)–bacDNA (10 μg ml^−1^) complexes, or by CXCL4 or DNA alone (same concentration) for 3 h, then cells were extensively washed (w). Subsequently, the pDCs were stimulated with bacDNA or CXCL4 (as above), and IFN-α production was measured by ELISA. Horizontal bars are the means, vertical bars are s.e.m., *P*-values by Student’s *t* test for paired samples (one-tailed)

**Fig. 4:**
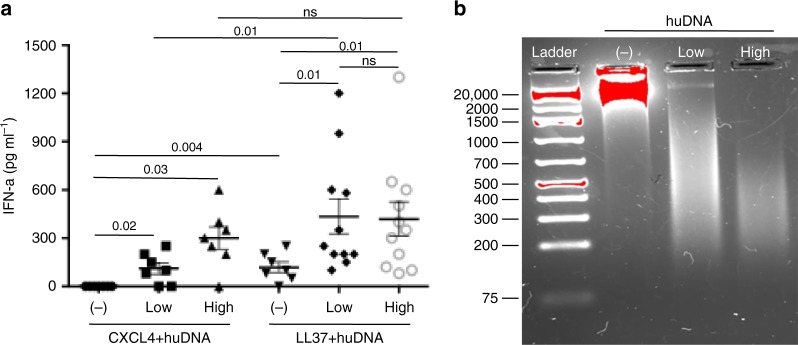
Stimulation of pDCs by CXCL4–DNA complexes is sensitive to the degree of DNA fragmentation. **a** PDCs were stimulated with huDNA unfragmented (−) or huDNA moderately (low) and highly (high) fragmented by sonication (see Methods) in complex with CXCL4 (1 µM) or LL37 (10 µM). IFN-α production was quantified by ELISA after 24 h. Horizontal bars are the mean, vertical bars are s.e.m., *P*-values from Student’s *t* test for paired samples (two-tailed). **b** Two percent agarose gel shows unfragmented (−) and the degree of fragmentation (low and high) of the huDNA preparation used for the functional assays depicted in **a**

SSc pDCs were more potent producers of IFN-α upon CpG DNA stimulation than controls, and CXCL4 acted synergistically with CpGs to induce IFN-α release by HD pDCs^3,4^. Although CpGs are mimics of bacterial DNA, they are artificial molecules designed to induce maximal TLR9 stimulation and chemically modified to resist degradation. In comparison, natural naked DNA is a much weaker TLR9 agonist^22^. Indeed, bacDNA alone only stimulated pDCs at high concentrations (30–100 μg ml^−1^), whereas huDNA was unable to induce IFN-α (Supplementary Fig. 5). CXCL4 interacting electrostatically with DNA might form immune complexes with subthreshold concentrations of DNA (<10 μg ml^−1^) and induce effective activation of pDCs. We assessed dose responses by varying CXCL4 concentrations incubated with fixed concentrations of bacDNA (Fig. 3a), and vice versa (Fig. 3b). We used LL37, which binds DNA and stimulates IFN-α release via TLR9 in pDCs, as a positive control^22^. While CXCL4 alone or bacDNA alone (10 μg ml^−1^) did not induce any detectable IFN-α secretion by pDCs, the complexes formed by CXCL4 and bacDNA strongly stimulated the cells (Fig. 3a, b). The doses of CXCL4 complexed with DNA inducing the strongest pDC-derived IFN-α were in the range of 3–12 μg ml^–1^ (between 0.5 and 2 μM) (Fig. 3a, b). Even in complexes with a very low dose of bacDNA (1 μg ml^–1^), CXCL4 induced significant IFN-α production (Fig. 3b). We treated pDCs with bacDNA alone or CXCL4 alone, sequentially adding CXCL4 or bacDNA, respectively. Sequential stimulation completely abrogated IFN-α release by pDCs, indicating that complex formation is essential to immune activation (Fig. 3c). Altogether, these results demonstrate that CXCL4 induces hyperactivation of pDCs and IFN-α production only when forming complexes with natural DNA. Complexes formed by CXCL4 and two preparations of huDNA fragmented by sonication (see Methods), which mainly contained fragments of 100–1000 base pairs (bp), induced significant IFN-α production (Fig. 4a, b). In comparison, CXCL4 complexed with unfragmented (unsonicated) huDNA (which contained mainly fragments of 2000/20,000 bp) was not stimulatory (Fig. 4a, b). This differed from LL37, which induced low but significant still significant IFN-α production not only when complexed with short DNA but also with long huDNA (Fig. 4a). Accordingly, CXCL4–bacDNA complexes inducing optimal pDC activation contained mainly small DNA fragments (100–700 bp) (Supplementary Fig. 6a, b), whereas CXCL4 bound to unfragmented bacDNA was significantly less stimulatory. Again, both highly fragmented and lowly fragmented bacDNA in complex with LL37 were equally effective at stimulating pDCs. These experiments indicate that the length and polydispersity of DNA fragments influence the stimulatory capacity of CXCL4–DNA more than that of LL37–DNA complexes. These data also show the potential of CXCL4 to break immune tolerance to self-DNA, providing that a certain degree of DNA fragmentation is present.

### CXCL4–DNA pDC activation is TLR9-dependent but not CXCR3-dependent

**Fig. 5:**
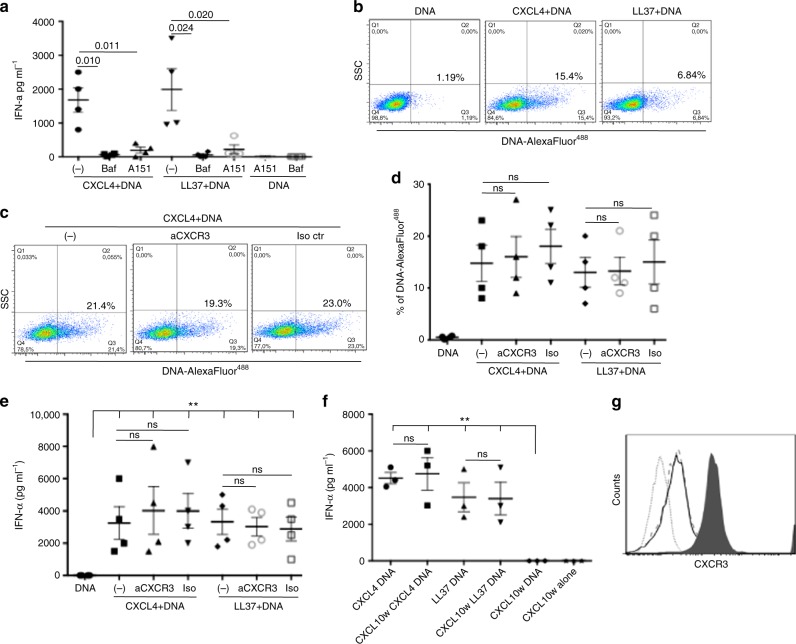
Activation of pDCs by CXCL4–DNA is TLR9-dependent and CXCR3-independent. **a** PDCs were stimulated with CXCL4 (1 μM)–DNA (10 μg  ml^−1^) or LL37(10 μM)–DNA (10 μg ml^−1^) complexes in the presence/absence of bafilomycin A (Baf), an inhibitor of endosomal acidification, or A151, a specific inhibitor of TLR9. Horizontal bars represent the mean, vertical bars s.e.m. *P*-values by Student’s *t* test for paired samples (one-tailed). **b** pDCs were treated with CXCL4 (1 μM) or LL37 (10 μM) in complex with 10 μg ml^−1^ of AlexaFluor 488-conjugated huDNA, or with huDNA alone. DNA entry into pDCs was quantitated by flow cytometry. One representative experiment out of five was performed with huDNA or bacDNA. **c** pDCs were treated with CXCL4–huDNA complexes in the presence/absence of a blocking anti-CXCR3 or an isotype control antibody and analyzed by flow cytometry. **d** Cumulative data on huDNA/bacDNA uptake into pDCs quantitated for CXCL4–DNA complexes in the presence/absence of neutralizing anti-CXCR3 (10 μg ml^−1^) or isotype control antibody as in **c**. Horizontal bars are the mean, vertical bars s.e.m. Significance by Student’s *t* test for paired samples (two-tailed). **e** PDCs were stimulated (bacDNA at 10 µg ml^−1^) as indicated, in the presence/absence of the blocking anti-CXCR3 (10 µg ml^−1^) or isotype control antibody (10 µg ml^−1^) and IFN-α production was measured by ELISA after 24 h. Horizontal and vertical bars represent the mean and s.e.m., respectively. Significance by the Student’s *t* test for paired samples (two-tailed). **f** PDCs were pretreated with CXCL10 (10 µg ml^−1^) for 1 h, washed, and stimulated with CXCL4–bacDNA or LL37–bacDNA complexes. IFN-α production was measured by ELISA after 24 h. Horizontal and vertical bars represent the mean and s.e.m. *P*-values calculated by Student’s *t* test for paired samples (two-tailed). **g** PDC expression of CXCR3 by flow cytometry after no treatment (filled histogram) or 2 -h treatment (and after washing) with anti-CXCR3 blocking antibody (10 µg ml^−1^) (unfilled histogram) or with CXCL10 (10 µg ml^−1^) (unfilled dashed histogram). Flow cytometry isotype control is shown as light gray dotted histogram

To investigate whether pDC activation was dependent on endosomal access and TLR9 binding, we pretreated pDCs with bafilomycin A1, an endosomal compartment acidificator or with ODN TTAGGG (A151), a TLR9 inhibitor. In both conditions, CXCL4–DNA complexes (Fig. 5a) and LL37–DNA complexes^22,25^ were unable to stimulate pDCs. Thus, trafficking to endosomal compartments and TLR9 engagement are required for immune activation by CXCL4-DNA complexes.

To assess whether CXCL4 could serve as a “molecular chaperone” for DNA delivery into pDCs, we exposed pDCs to fluorescently-labeled DNA alone or complexed with CXCL4 to track DNA entry into pDCs: DNA was internalized by pDCs only when complexed with CXCL4 (Fig. 5b), or when complexed with positive control LL37^22^.

CXCR3 is an important signaling receptor for CXCL4^14,26^. We investigated whether CXCR3 is required for binding/internalization of CXCL4–DNA complexes. Fresh pDCs consistently expressed CXCR3^24,26^, but CXCR3 blockade by a neutralizing specific antibody did not inhibit internalization of CXCL4–DNA complexes (Fig. 5c, d cumulative data). Accordingly, CXCR3 blockade did not affect the ability of CXCL4–DNA complexes to induce IFN-α production, suggesting that pDC activation by CXCL4–DNA complexes is CXCR3-independent (Fig. 5e). Moreover, saturation of CXCR3-binding sites with the CXCR3-binding chemokine CXCL10^27^ did not inhibit IFN-α production by CXCL4–DNA complexes (or LL37–DNA complexes) either (Fig. 5f). Both anti-CXCR3 antibodies or treatment with CXCL10 strongly reduced CXCR3 surface expression in pDCs (Fig. 5g), further confirming that the presence and/or high-level expression/availability of CXCR3 receptors were not required for the stimulatory activity of CXCL4–DNA complex pDC-IFN-α release. These blocking experiments confirmed that CXCL4–DNA complexes stimulated pDCs by binding to endosomal TLR9, and unexpectedly ascertained that internalization of, and stimulation by, CXCL4–DNA complexes is CXCR3 independent.

### CXCL4–DNA forms TLR9-tailored liquid crystalline complexes

**Fig. 6:**
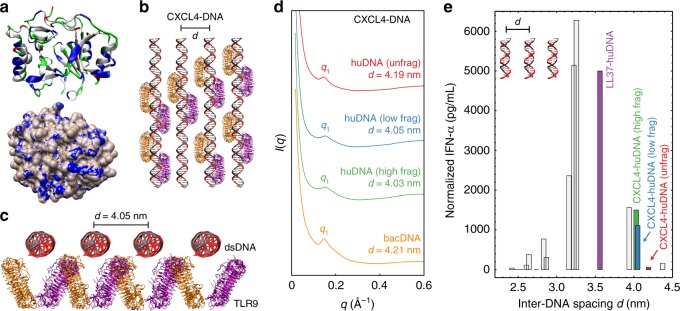
CXCL4 organizes both self-DNA and bacDNA into liquid-crystalline supramolecular complexes at an inter-DNA spacing that facilitates amplified TLR9 activation. **a** Structure of CXCL4 (PDB ID: 1F9Q) with solvent-exposed putative DNA-binding cationic surface residues in blue and hydrophobic residues in white (top). Uncharged polar residues are shown in green and anionic residues are shown in red. Molecular structures were visualized in visual molecular dynamics (VMD). TLR9 structure (PDB ID: 3WPG) was obtained from previous work^28^. The electrostatic potential map of the CXCL4 surface was calculated in Chimera using the APBS plugin (bottom). Blue corresponds to a positive electrostatic potential and white corresponds to a neutral potential. **b** Graphical schematic to scale of ordered CXCL4–DNA complexes binding to clustered TLR9. CXCL4 is omitted for clarity. Top–down view and **c** end-on view of CXCL4-fragmented huDNA complexes binding to TLR9 dimers (orange and purple). CXCL4 organizes huDNA (black–gray) into a liquid-crystalline columnar complex with an average inter-DNA spacing *d* = 4.05 nm, near the optimal value for intercalative multivalent binding to a clustered array of TLR9 receptors, consistent with elevated levels of IFN-ɑ production. **d** SAXS data of CXCL4 bound to huDNA and bacDNA: The first peak positions *q*_1_ and inter-DNA spacings (*d*) are indicated. CXCL4 forms liquid-crystalline supramolecular complexes with huDNA and bacDNA, cognate to those formed with LL37 but with a lower degree of DNA ordering (Supplementary Fig. 7). **e** Plot of TLR9 activation vs. inter-DNA spacing for CXCL4–huDNA complexes. The fragmentation state of huDNA affects the ability of complexes to induce IFN-ɑ production from pDCs. Gray bars derived from previously published data^28^. Relative TLR9 activation levels are consistent with the relative levels of cytokine production predicted from the inter-DNA spacings

Next, we investigated the structural basis for CXCL4-enabled immune amplification via TLR9. CXCL4 is a hybrid α-helical/β-sheet molecule with a net cationic charge (+3.1 at pH 7.4)^14^. Secondary structure analysis revealed several cationic/amphipathic helices arranged around its circumference, potentially binding anionic DNA (Fig. 6a, top). Calculation of the electrostatic surface potential of CXCL4 uncovered several solvent-exposed cationic surfaces for multivalent interactions with the DNA phosphate backbone (Fig. 6a, bottom). This suggests that CXCL4 has the physicochemical requirements to bind electrostatically to DNA. However, CXCL4–DNA might form disordered aggregates, ordered crystals, and adopt liquid crystalline structures between these extremes. Rather than optimizing crystallization conditions to solve the structure of a CXCL4–DNA crystal, we used synchrotron SAXS to directly examine CXCL4–DNA structures in solution, to more appropriately mirror the natural state of CXCL4 in SSc tissues/blood. SAXS measurements were performed on equilibrium phase-separated liquid-crystalline CXCL4–DNA complexes in aqueous solution, mimicking plasma conditions (140 mM NaCl + 10 mM HEPES, pH 7.4, 37 °C).

We demonstrated previously that TLR9 activation in pDCs is strongly influenced by the inter-DNA spacing “*d”* between parallel DNA ligands in complexes formed by DNA with AMPs^28–31^. Specifically, inter-DNA spacing close to the steric size of TLR9 (*d* = 3–4 nm) allows optimal multivalent binding of columnar DNA lattices to TLR9-clustered arrays. The degree of crystallinity in such supramolecular complexes also influenced the activation of TLRs^28–31^. Consequently, we measured the crystallinity parameters of inter-DNA spacing *d* of CXCL4–DNA complexes (Fig. 6b, c, details in Methods section) to compare them to known TLR9-activatory supramolecular complexes.

We found that CXCL4 organizes DNA into liquid-crystalline columnar lattices at an inter-DNA spacing compatible with TLR9 amplification (Figs. 6b, c). We first solved the structures of CXCL4 bound to unfragmented huDNA. A DNA correlation peak was measured at *q*_1_ = 0.150 Å^−1^, consistent with an inter-DNA spacing of 4.19 nm (Fig. 6d), which is in the range where amplification of TLR9 activation is observed^28,29^. By way of comparison, LL37 organizes DNA into square columnar lattices with a more optimal inter-DNA spacing of *d* = 3.56–3.57 nm (*q*_1_ ~0.176 Å^−1^) with both bacDNA and huDNA as shown^28^, and of 3.34–3.37 nm with more fragmented huDNA (Supplementary Fig. 7). These mutually consistent results indicate that CXCL4–DNA complexes are expected to amplify TLR9 responses, in a manner cognate to LL37–DNA complexes.

The degree of DNA fragmentation can affect the crystallinity of structures formed with CXCL4. With fragmented huDNA, CXCL4 formed columnar lattices with slightly smaller inter-DNA spacings (*q*_1_ = ~0.156 Å^−1^, *d* = 4.03–4.05 nm) (Fig. 6d). This smaller spacing (fitting the size of TLR9 better, being closer to the optimal *d* of 3–4 nm) is in agreement with the finding that CXCL4 bound to fragmented huDNA induced IFN-α more efficiently than CXCL4 bound to unfragmented huDNA (Figs. 4, 6e). We observed that CXCL4–huDNA complexes induced IFN-ɑ production by pDCs consistent with levels predicted from the measured inter-DNA spacing (Fig. 6e). Interestingly, the decreased inter-DNA spacing in CXCL4-fragmented huDNA complexes is closer to the value that produces maximal pDC activation. This can lead to increased binding of clustered DNA to TLR9, potentially explaining why higher IFN-α production is expected and observed with sonicated huDNA. Physically, fragmentation of DNA increases the total number of discrete DNA fragments for the same mass of DNA with more optimal close packing of DNA ligands. “Hairpin” packing defects, necessary to organize longer DNA polymers into lattices, may elastically mitigate against closer crystalline ordering.

The hydrodynamic sizes of the CXCL4–DNA complexes, as measured by dynamic light scattering (DLS) for fragmented/unfragmented DNA were about ~1 μm, larger than that for compact LL37–DNA complexes (~0.25 μm), but well in the range that renders them amenable for endocytosis into immune cells^32^. Using methods similar to those that we and others used for biomolecular systems^33,34^, the domain sizes of ordered domains from CXCL4–DNA complexes are typically <10 nm, and are smaller than those for LL37–DNA complexes^35^. This suggests the intriguing possibility that CXCL4–DNA complexes have small ordered immunogenic domains with ideal inter-DNA spacings for TLR9 activation embedded within larger disordered complexes. Altogether, the SAXS data indicate that CXCL4 organizes huDNA and bacDNA into immunogenic liquid-crystalline complexes suitable for TLR9-mediated IFN-α production.

### PDC stimulation by SSc blood-derived CXCL4 is DNA dependent

**Fig. 7:**
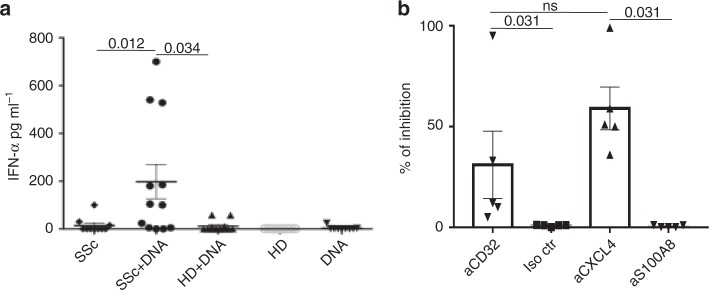
CXCL4 from SSc blood stimulates pDCs in a DNA-dependent manner. **a** PDCs were stimulated with SSc plasma alone (SSc, 1:50 dilution) pretreated with 10 µg ml^−1^ of huDNA (SSc+DNA) or with plasma from HD (same dilution) also pretreated with huDNA (HD+DNA) or untreated (HD), or with huDNA alone (DNA). IFN-α was quantitated by ELISA after 24 h. Horizontal bars are the means, vertical bars are the s.e.m. *P*-values by Wilcoxon signed-rank test. **b** PDCs were treated with SSc plasma pretreated with huDNA as in **a** in the absence or in the presence of a neutralizing anti-CXCL4 antibody (aCXCL4; see Methods), neutralizing anti-CD32 antibody (aCD32)^16^, corresponding control (Iso ctr), or irrelevant antibodies (aS100A8), and IFN-α was analyzed after 24 h by ELISA. Data are expressed as percent of inhibition of IFN-α production (using as a reference the amounts of IFN-α obtained after stimulation with SSc plasma pretreated with huDNA). Significance was assessed by Wilcoxon signed-rank test

Structural and functional data on CXCL4–DNA complexes were obtained using three different preparations of human CXCL4 (see Methods). To determine whether natural-plasma-derived CXCL4 behaves similarly, we stimulated HD pDCs with SSc plasma that contained high levels (195,000 ± 72,106 pg ml^−1^) or with HD plasma that contained low levels of CXCL4 (134 pg ml^−1^ ± 35) (Fig. 7a). The addition of huDNA to SSc plasma, and not to HD plasma, significantly increased IFN-α production (Fig. 7a), an effect blocked by anti-CXCL4^3^, but not by control anti-S100A8 antibodies (Fig. 7b). Since it was previously shown that SSc plasma/sera have interferogenic effects on pDCs in vitro due to the presence of autoantibodies (anti-scleroderma 70—Scl-70 or anti-centromere—ACA), we used a blocking anti-CD32 antibody to inhibit antibody-mediated pDC activation/IFN-α release^8,9,23^. A blocking anti-CD32 antibody, unlike an isotype-matched control, significantly inhibited IFN-α release by pDCs stimulated with huDNA pretreated plasma, indicating IFN-inducing effects of autoantibodies, as previously demonstrated^8,9^ (Fig. 7b). However, we were unable to detect a significant correlation between levels of Scl-70 and IFN-α in the discovery (*r* = −0.06, *P* = 0.38, *N* = 25) and replication (*r* = 0.03, *P* = 0.4, *N* = 52) SSc cohorts. Further, ACA-positive and ACA-negative patients did not differ in their mean plasma/serum IFN-α (13.3 vs. 13.1 pg ml^−1^, *P* = 0.28, *N* = 25, discovery SSc cohort; 398 vs. 403 pg ml^−1^, *P* =0.1, *N* = 49, replication SSc cohort). These experiments indicate that blood-derived CXCL4, besides autoantibodies, represents an important component of the IFN-α production machinery in the presence of self-DNA.

### CXCL4–DNA complexes are present in SSc blood and tissues

**Fig. 8:**
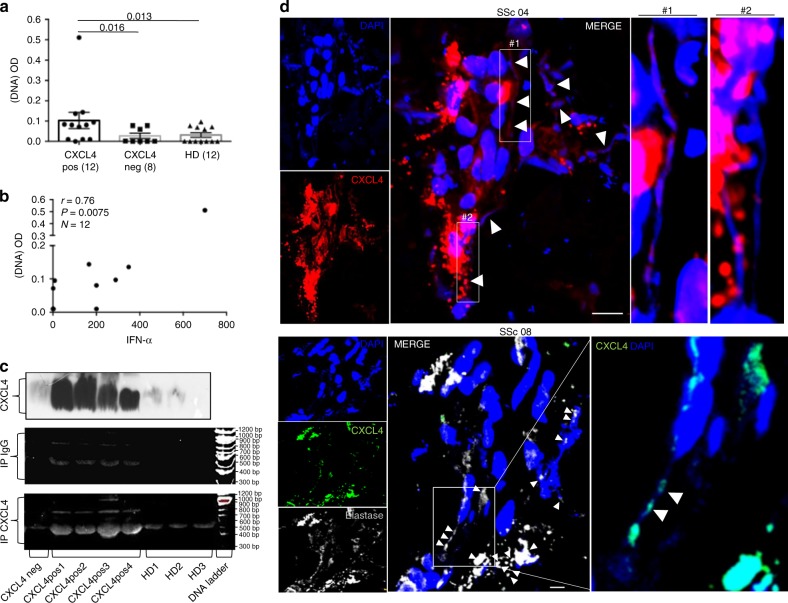
CXCL4–DNA complex detection in vivo. **a** SSc plasma that was either positive (SSc pos, *N* = 12) or negative (SSc neg, *N* = 8) for CXCL4 content and HD plasma (that were all negative for CXCL4 content, *N* = 12) were plated on 96-well plates coated with a mouse anti-CXCL4 antibody. After washing, levels of CXCL4–DNA complexes were measured by using an anti-dsDNA antibody (see Methods, 1:300 dilution). Results are expressed as optical density (OD). Horizontal bars are the mean, vertical bars are s.e.m., *P*-values by Mann–Whitney test. **b** CXCL4–DNA immune complexes of CXCL4-positive patients (measured as in panel **a**) were plotted against IFN-α plasma levels to assess correlation between the presence of circulating CXCL4–DNA complexes and IFN-α in SSc patients (*N* = 12). Spearman’s correlation coefficient “*r*”, significance “*P*”, and sample size “*N*”, were indicated. **c** CXCL4 (upper panel) was immune-precipitated using an anti-CXCL4 antibody from plasma of SSc patients that were negative (SSc neg, *N* = 1) or positive (SSc pos, *N* = 4) for CXCL4 content, and from HD plasma (*N* = 3). The immune-precipitated material was run on a gel (8% acrylamide) and DNA was stained by ethidium bromide. In the middle panel, ethidium bromide staining of the same SSc and HD plasma immune-precipitated for IgG–immune complexes (IP IgG, see Methods); in the lower panel, ethidium bromide staining of plasma immune-precipitated for CXCL4 (IP CXCL4). Results are representative of one experiment of two performed, with different SSc and HD plasma. **d** LSM images relative to the presence of extracellular traps in skin biopsies from two different SSc patients out of four that show DNA filaments (blue, DAPI), CXCL4 in red (upper panels, patients SSc 04), or in green (lower panels, patient SSc 08), elastase in gray (lower panel) (magnification ×63; bar, 5 µm). CXCL4–DNA complexes are indicated by white arrowheads; high-power images of some of the indicated complexes are provided as insets for both patients. Results from two biopsies of eight analyzed

We used complementary approaches to demonstrate the presence of CXCL4–DNA complexes in vivo. First, we coated ELISA plates with anti-CXCL4 antibody to capture circulating CXCL4, and then added anti-dsDNA antibody to detect dsDNA/CXCL4 circulating complexes. The SSc plasma was divided into CXCL4-positive (*N* = 12, SSc pos) and CXCL4-negative (*N* = 8, SSc neg) subsets. CXCL4-positive plasma gave a positive signal for dsDNA in ELISA in 5 of 12 (42%) CXCL4-positive plasma (Fig. 8a). All control SSc/HD plasma negative for CXCL4 content were negative for dsDNA. We found a positive, significant correlation between plasma IFN-α levels and positivity for dsDNA (*r* = 0.76, *P* = 0.0075, *N* = 12), indicating correlation between blood IFN-I-signature and the presence of circulating CXCL4–DNA complexes (Fig. 8b). Analysis of the sole eaSSc patients (*N* = 9) showed a stronger correlation between plasma IFN-α levels and circulating CXCL4–DNA complexes (Spearman’s *r* = 0.87, *P* = 0.002). Second, to visualize circulating CXCL4–DNA complexes, we immune-precipitated plasma CXCL4 (Fig. 8c, upper panel) and ran the immune-precipitated CXCL4 on a gel (Fig. 8c, lower panel), revealing bound DNA by ethidium bromide staining. Considerable amounts of DNA were immune-precipitated from SSc CXCL4-positive plasma, and not from CXCL4-negative, control plasma (Fig. 8c, lower panel). As a control, we also immune-precipitated plasma antibody–immune complexes (IP IgG), detecting a slight reactivity with ethidium bromide, indicating that DNA partially bound to IgG–immune complexes (Fig. 8c, middle panel). Interestingly, DNA bound to CXCL4 was mainly of a fragment length between 400 and 1000 bp, equivalent to the DNA size optimal for TLR9-induced IFN-α production in pDCs. Third, we assessed whether CXCL4 could be associated with NET-like structures in SSc skin. The granulocyte markers myeloperoxidase (MPO) and elastase were detected in all nine (100%) SSc skins by LSM, although the staining intensity was highly variable among patients (Supplementary Fig. 8). High magnification of LSM images revealed NET-like structures (filaments of DNA) in four of eight skin biopsies (50%) analyzed (Fig. 8d). CXCL4 decorated the DNA filaments, which mostly arose from elastase-positive cells (Fig. 8d, lower panels)^36–38^.

Altogether, these data indicate that CXCL4–DNA immune complexes exist both in circulation and SSc-affected tissues, which may enable CXCL4 to really exert its adjuvant activity in vivo.

## Discussion

In this study, we discovered how CXCL4, a molecule overexpressed in SSc, in several other chronic conditions^39–42^, and during physiological immune responses to infections/trauma, drastically amplifies IFN-I production by breaking immune tolerance to self-DNA. We identify a novel pathway in which CXCL4 organizes DNA into liquid-crystalline immune complexes with inter-DNA spacings optimal for TLR9 amplification. Systematic investigation of the interactions between CXCL4–DNA complexes and TLR9 suggests that the central paradigm for molecular recognition of immune ligands acting as pathogen-associated/damage-associated molecular patterns (PAMPs and DAMPs^43,44^) by immune receptors needs to be generalized. Indeed, we find that TLR9 recognizes and responds not simply to patterns of individual DNA molecules but to the different supramolecular co-ordering of the DNA with specific chaperone molecules^29–31^. This is all the more surprising, given that TLR9 receptors are thought to bind to single molecules of dsDNA rather than partially ordered, liquid-crystalline arrangements of dsDNA. It is well known that CXCL4 binds heparin and other anionic molecules with similar/opposite charge densities^14,45,46^. What is surprising and salient in terms of immune outcomes is that CXCL4 can behave as an effective “adjuvant” by ordering DNA into a pro-inflammatory supramolecular structure. The capacity of CXCL4 to chaperone nucleic acids into pDCs is not the only requirement to deliver tissue damage signals: chaperones must also organize DNA ligands into the right-ordered supramolecular liquid crystals, which effectively induce clustering of TLRs and subsequent immune amplification^22–25,29–31,47^. In this respect, we show for the first time that CXCL4 is both a chaperone, because it protects DNA from degradation and facilitates internalization of DNA into pDCs, and an adjuvant because it renders DNA hyperstimulatory for TLR9.

Interestingly, the potency of the CXCL4 adjuvant activity depends on the polymeric structure of DNA, in this case by DNA length. Under physiological conditions, DNA released by dying cells is extensively fragmented by nucleases. It is therefore counterintuitive that degradation of self-DNA, in the presence of CXCL4, could actually lead to exacerbation of a TLR9-mediated inflammation and disease. The existing paradigm assumes that DNA degradation abolishes its immunological potential^48^, as shown in animal models of SLE. Our experiments imply that DNA released by dying cells and partially fragmented by nucleases can be rendered more immunogenic when assembled into supramolecular complexes by CXCL4, which acts as an adjuvant in vivo. (Clearly, we do not expect activation in the extreme limit of complete enzymatic degradation.) However, the degree/rate of the DNA fragmentation may depend on the type of cell death that occurs in the inflammatory disease, and accessibility of DNA to nucleases, with necrosis or pyroptosis more prone to releasing free DNA, accessible to degrading enzymes. SAXS analysis found that complexes formed between CXCL4 and more fragmented huDNA (which corresponds to shorter average DNA lengths and greater polydispersity degree) lead to liquid-crystalline ordering with a smaller inter-DNA spacing that matches the steric size of TLR9 slightly better. Although our results are consistent with the observation that complexes of fragmented huDNA with CXCL4 are activating, whereas those formed with unfragmented huDNA are not, we recognize that it is likely that effects, such as differential endosomal access due to size, can also contribute. The observed dependence on DNA length and polydispersity of CXCL4–huDNA complex stimulatory ability represents a significant challenge to traditional explanatory mechanisms. Indeed, this is difficult to reconcile with a simple molecular recognition where single dsDNA molecules bind to single TLR9 receptors, which should not be impacted by the DNA length. The findings here suggest that different adjuvants, by enforcing distinct forms of supramolecular ordering of immune ligands, may induce differential TLR responses. In this way, innate immune cells can react to a much wider range of challenges than those previously anticipated in the PAMP/DAMP paradigm^29–31,43,44^ and innate immune receptors can reach multiple levels of sensitivity.

To date, only a few endogenous molecules have been shown to break immune tolerance to self-DNA^22–25,47^, as shown in SLE and psoriasis, via nucleic acid binding and TLR-dependent dendritic cell hyperactivation^22–25^. It is interesting to compare CXCL4 to the known adjuvants LL37 and HBD3, which are prototypical antimicrobial peptides^22,23,25^. Although present in circulation, LL37 is produced mostly at epithelial surfaces, and is released by neutrophil degranulation or by generating NETosis at inflammation sites^23^. HBD3 expression is even more tissue specific^20,21^. As a result, the adjuvant effects of HBD3 and LL37 are most likely confined to epithelial surfaces. In contrast, large amounts of CXCL4 are released by activated platelets^14^, which are considered important mediators of SSc pathogenesis and fibrosis induction^36^. Thus, the amount of available CXCL4 may be an important variable to explain CXCL4 adjuvant activity specifically in SSc. We and others found that CXCL4 reaches very high concentrations in both the SSc-affected skin and the blood^3,4^, especially in early SSc. Notably, an IFN-I-gene signature early in disease correlates with cardiovascular manifestations, lung fibrosis, and an overall poor prognosis^8,9,12^. Thus, formation of CXCL4–DNA complexes, as an early event, may exacerbate disease. Therefore, a crucial issue is whether CXCL4–DNA immune complexes really exist in vivo in SSc. We show that such complexes are present in SSc circulation and tissues and we speculate that the CXCL4-bound DNA originates from cells dying in SSc-affected tissues or in blood vessels, or derives from activated plateles^14^. A striking finding is that the presence of circulating CXCL4–DNA complexes consistently correlates with IFN-I signature in blood, and this correlation increases in early SSc. The visualization of CXCL4 decorating DNA filaments resembling NET-derived DNA in SSc tissues is in accordance with a recent study, showing that CXCL4 can bind NET–DNA in vitro^37^. A recent paper suggests a role for neutrophils and platelet-induced NETosis in SSc pathogenesis^36^. In our hands, neutrophil elastase-expressing cells were the source of DNA filaments. All granulocytes, but also activated macrophages^38^, which consistently infiltrate SSc skin, express neutrophil elastase and all these cells, and mast cells, can undergo a process similar to NETosis, generally referred to as ETosis (extracellular trap release)^49^. Thus, it is presently unclear which cell type is the main source of extracellular DNA in the SSc skin.

Pre-analytical sample preparation can be critical when assessing CXCL4 levels, since platelet activation during clotting may result in CXCL4 release. We used plasma in our ex vivo determination of the CXCL4 markers/effector functions to minimize such a problem. However, both in our discovery cohort with plasma and the replication SSc cohort using sera, we observed higher levels of CXCL4 than in controls, and a significant and specific correlation between CXCL4 and IFN-α levels, strongly supporting our general conceptual framework.

CXCL4 may not be the only molecule that contributes to the IFN-I signature observed in SSc. LL37 itself, used as reference molecule through this study, is expressed in SSc skin and is a further candidate contributor to the IFN-I signature in the SSc skin^19^. However, in SSc, blood concentrations of LL37 were clearly not elevated and in our SSc cohort, circulating LL37 levels did not correlate with IFN-α levels. Furthermore, serum levels of S100A8 and S100A9 were very high in SSc as reported^20^, but they did not correlate with IFN-α levels either, consistent with the inability of S100A8 and S100A9 to bind DNA and stimulate pDCs. Importantly, immune complexes formed by SSc-associated autoantibodies were shown to stimulate pDCs in a TLR9-dependent manner and induce IFN-α^8,9^. We reproduced this in our in vitro experiments and we also visualized some DNA bound to immune-precipitated IgG complexes (which can be classical or previously uncharacterized SSc autoantibodies). However, CXCL4 bound higher amounts of DNA than IgG complexes, and bound DNA possessed the optimal size for TLR9 stimulation.

Importantly, our study is relevant not only for SSc^3,4^ or other chronic conditions, but also for the role of CXCL4 in normal immune responses to infections/traumas in which platelets, as the major source of CXCL4, exert crucial roles in immunity and tissue repair^50^. Of note, IFN-I activates wound healing, a process underlying fibrosis^51,52^. However both CXCL4, via binding to its receptor CXCR3, and IFN-I itself, mainly inhibit angiogenesis^14,53^. New blood vessel formation is critical for wound healing; thus, the CXCL4–DNA complex effect on endothelial cells and fibroblasts warrants further investigation to clarify the role of the complex in tissue repair. CXCL4 is also an antimicrobial molecule active against bacteria and viruses^54,55^. Thus, the ability of CXCL4 to induce IFN-I may have importance in controlling viral infections. The strongest adjuvant effect obtained with CXCL4-bacDNA complexes reinforces the role of CXCL4 as a crucial antibacterial agent^14^. IFN-I is beneficial during infections by extracellular bacteria^54,55^ and contributes to differentiation of B cells into antibody-secreting plasma cells^56^.

In conclusion, we demonstrated that the immunomodulatory role of CXCL4 in physiological/pathological conditions is not limited to its canonical signaling function via CXCR3, but rather includes an unexpected ability to organize DNA into strongly immunomodulatory liquid-crystalline supramolecular complexes. These striking findings suggest that disrupting CXCL4 adjuvant activity could represent a therapeutic opportunity in SSc, especially if cell types other than pDCs are also stimulated by complexes formed by CXCL4 and nucleic acids^4,57–60^.

## Methods

### Patients

This study was approved by the ethical committee of the institutions involved (“Commission cantonale d’éthique de la recherche”, Geneva, Switzerland; the Ethics Committee of the Sapienza University and Tor Vergata University, Rome, and University Hospital of Bordeaux, France) and was conducted according to the Declaration of Helsinki. Informed, written consent was obtained from all participants according to the declaration of Helsinki. Patients with SSc satisfied the ACR/EULAR 2013 classification criteria for SSc^61^ and their clinical presentation was defined according to LeRoy et al.^62^. Their clinical characteristics are detailed in Supplementary Table 1. Skin biopsies were performed on the affected skin of SSc individuals or on age-matched and sex-matched healthy individuals undergoing corrective plastic surgery (Department of Plastic Surgery, Geneva University Hospitals, Switzerland). Blood samples (sera and plasma) were collected from SSc patients at Policlinico Umberto I, University La Sapienza, Rome, Italy. Sera from patients with SLE, used as a control, were obtained from the Swiss SLE Cohort Study^63^. Samples from sex-matched and age-matched HD were obtained from the blood center of “University La Sapienza, Policlinico Umberto I”, Rome, Italy and from “Centre de transfusion sanguine, Hopitaux Universitaires de Genève”, Switzerland.

### Reagents

LL37 was from Innovagen (Sweden) or Proteogenix, (France). S100A7 (psoriasin) was from Abcam. CXCL4 was from SINO Biological (China) or Novus Biological (UK) or Biomatik (Italy). S100A8 and S100A9 were from SINO Biological; human genomic DNA (huDNA) was from BioChain (San Francisco, CA, USA) or Roche (CH). BacDNA (*E. coli*) was from Invivogen (San Diego, CA, USA); Bafilomycin A was from Sigma Aldrich (USA) and was used at 50 nM. BacDNA was also isolated from bacteria *E. coli* (strain DH5a) cultured in Luria Broth (Conda), while huDNA was also extracted from peripheral blood mononuclear cells (PBMCs) (from buffy coats, see below) by using the Qiagen kits mericon DNA Bacteria Kit and DNA blood Maxi Kit (Germany). CXCL10 (IP10) was purchased from PeproTech (London, UK). The TLR9 inhibitor ODN TTAGGG (A151) was purchased from Invivogen and used at a concentration of 3 µM.

### DNA fragmentation

HuDNA or bacDNA preparations, purchased or extracted from PBMCs or *E. coli* cultures, respectively (as above), were fragmented by sonication. Five milligrams of DNA in a volume of 300 µl were fragmented by using the Sonics Vibra Cell sonicator (Sonics & Materials Inc.), with the following settings: 2, 4, and 10 sonication cycles (30 s ON, 30 s OFF in ice) to obtain the DNA fragment size between 100 and 1000 bp. The resulting size distribution was controlled by 2% agarose gel electrophoresis.

### Isolation/stimulation of blood pDCs

For isolation of human peripheral blood pDCs, blood buffy coats of healthy donors (HD) were obtained from “Centre de Transfusion sanguine of Hopitaux Universitaire”, Geneva, CH, and Blood Center of Policlinico Umberto I, Rome, IT. After separation of PBMCs by Ficoll centrifugation, pDCs were purified as described^23,41^ by using Diamond Plasmacytoid Dendritic Cell Isolation Kit (Miltenyi Biotec). Cell purity was evaluated by staining the cells with anti-CD123-APC and anti-HLADR-PerCPcy5.5 antibodies (both from BD Pharmingen) and flow cytometry acquisition (Supplementary Fig. 9). Purified pDCs were seeded into 96-well round-bottom plates at 300 × 10^3^ cells ml^−1^. LL37, S100A8, S100A9, psoriasin, and CXCL4 were premixed with total huDNA or bacDNA (10 µg ml^−1^ or, in some experiments 3 or 1 µg ml^−1^) and added to the pDC cultures after 15-min incubation at room temperature. Stimulation of pDCs was also performed with CXCL4–DNA or LL37–DNA complexes or plasma (1:50 dilution) or with huDNA-pretreated plasma in the presence of the following antibodies: anti-human-CXCR3 (R&D Systems, Minneapolis, MN, clone 49801 at 10 µg ml^−1^); control mouse IgG1 isotype control (10 µg ml^−1^, R&D); neutralizing rabbit polyclonal anti-human-CXCL4^3^ at 10 µg ml^−1^ and a control antibody directed to S100A8 (anti-S100A8/MRP-8 polyclonal rabbit, Merck Millipore, Germany); neutralizing anti-CD32 antibody^16^ (Abcam, AT10, 5 µg ml^−1^) and the related mouse monoclonal IgG isotype control (5 µg ml^−1^, Abcam). Antibodies were added to the pDC cultures 30 min before adding the complexes to discriminate the pathway of activation of pDCs.

### Measurement of proteins by ELISA

Supernatants of stimulated pDCs were collected after overnight culture and tested by using ELISA kits for detection of human IFN-α (MabTech, Sweden). The same ELISA was used to measure IFN-α in HD and SLE patients, after a dilution of 1:4 in PBS. To measure CXCL4 and S100A8/9 heterodimers in sera/plasma of SSc patients and control HD and SLE, we used the Human CXCL4/PF4 ELISA and the Human S100A8/S100A9 Elisa Duo set Kits from R&D System, respectively (after a pre-titration experiment, usually using dilution of 1:200, 1:100, or 1:50 in dilution medium according to each ELISA’s instruction). To measure LL37, we used the Human antibacterial peptide LL37 ELISA Kit (Cusabio, China).

### Measurement of SSc-specific antibodies in sera and plasma

Measurement of ACA in SSc sera/plasma was semiquantitative and was assessed by indirect immune fluorescence on HEp2 cells according to the protocol of the manufacturer (Menarini, IT). Measurement of anti-Scl70 antibodies in SSc sera/plasma was performed by a standard ELISA kit (Menarini).

### Measurement of CXCL4–DNA complexes

CXCL4–DNA complexes were identified using a capture ELISA. Capturing antibody, 2 µg ml^−1^ of mouse anti-human CXCL4 antibody (from Human CXCL4 DuoSet Elisa, R&D Systems) was coated to 96-well plates (100 µl) overnight at room temperature. After blocking in PBS 1% BSA (200 µl), plasma (100 µl diluted to 1:100 in 1% BSA in PBS) was added and incubated for 2 h at RT. After incubation, wells were washed three times with 200 µl of 0.05% Tween 20 in PBS, and the HRP-conjugated anti-dsDNA (Life Technologies, clone HpS22) was added for 1 h at room temperature. After washing, the chromogenic substrate 3.3’, 5.5’-tetramethylbenzidine (TMB) was added and incubated in the dark; the absorbance was measured at 450 nm after stopping the reaction by 2 N HCl. Plasma were considered positive when OD was above an established cutoff, which was calculated as the mean plus two times the standard deviation of OD values obtained with HD plasma.

### Visualization of protein-bound DNA by western blot

After preclearing for 3 h, plasma samples were incubated with anti-CXCL4 antibody ab9561 (Abcam), or IgG previously conjugated with Dynabeads protein G (Invitrogen), for 3 h. The immunoprecipitated immune complexes (IP) were washed three times with RIPA buffer (Sigma). For CXCL4 and IgG detection, the IP were loaded and run on a denaturing SDS/PAGE gel (15% of polyacrylamide) and blotted onto a nitrocellulose membrane (BioRad), which was incubated with the appropriate antibody (anti-CXCL4 or anti-human IgG, Sigma), and developed with the ECL system (Amersham Pharmacia Biotech). For detection of CXCL4–DNA and IgG–DNA complexes, IP were run into a polyacrylamide gel (8%), which was then stained with ethidium bromide.

### Hematoxylin and eosin staining

Paraffin sections dewaxed in xylene and hydrated through graded ethanols to deionized water were rinsed in PBS and stained with hematoxylin for 10 min, rinsed in tap water, then stained with eosin for 2 min, and rinsed in tap water and mounted.

### Binding of CXCL4 to DNA by PicoGreen assay

HuDNA or bacDNA were premixed with CXCL4 and control molecules at different protein–DNA ratios in a small volume (50 µl) and analyzed by a fluorimeter after staining with PicoGreen (Quant-iT PicoGreen dsDNA kit, Invitrogen), according to the standard protocol provided by the manufacturer. Samples were excited at 480 nm, and the emission intensity was measured fluorometrically at 520 nm.

### Electrophoretic mobility shift assays (EMSA)

Plasmid mixtures containing 150 ng (1.97 nM) of linear pDB29 DNA 5.7-kb plasmid^64^ in DPBS with or without 0.5 mM MgCl_2_ and 0.9 mM CaCl_2_ were incubated with CXCL4 or control molecules for 30 min at 37 °C.

Samples were run at 3 V cm^–1^ on 0.8% agarose gel in 1× TBE for 1 h at 4 °C, stained with ethidium bromide, and visualized under UV light (see below).

EMSA with huDNA was performed by mixing various μM concentrations of CXCL4 or LL37 or other control AMPs with appropriate concentration of huDNA (the same that induced IFN-α production in pDCs) and respecting the appropriate protein–DNA ratio used in pDC-stimulation assays. DNA alone and the mixtures were run on a 2% agarose gel to evidence the delay in migration of the DNA due to the binding to the tested proteins. HuDNA on the gel was visualized by coloring with SYBR Safe DNA gel staining (ThermoFisher Scientific).

### Restriction protection assay

Restriction assay solution (DPBS with 0.5 mM MgCl_2_, 0.9 mM CaCl_2_, and 0.1 mg/ml BSA) containing 150 ng (1.97 nM) of circular pDB29 DNA was incubated for 30 min at 37 °C with various concentrations of CXCL4 or control molecules in the presence/absence of 0.55 U of EcoRV enzyme (ThermoFisher Scientific). DNA products were extracted with phenol/CHCl_3_, ethanol precipitated, and electrophoresed on a standard 1% agarose gel in 1× TAE with ethidium bromide. The gel was pictured under UV light using a Chemi Doc MP system (Bio-Rad Laboratories) and unsaturated images were analyzed by densitometry using MultiGauge (v.3, Fujifilm LifeScience). As EcoRV cuts only once in pDB29, the percentage of digested DNA was calculated by dividing the density of linear DNA by the total DNA density (linear + relaxed–circular + supercoiled–circular) and multiplying by 100.

### Nuclease protection assay of huDNA and bacDNA

The suspension containing CXCL4–huDNA or CXCL4–bacDNA complexes (or complexes of DNA with control molecules) was incubated at 37 °C with 100 U ml^−1^ DNAse I (Roche, CH) for 10–30 min at 37 °C. Then, the DNA was stained with PicoGreen and quantitated by a fluorimeter at different time points (as above).

### DNA labeling by a fluorochrome and CXCL4–DNA visualization

HuDNA or bacDNA was labeled with Alexa Fluor 488 by using the Ulysis Nucleic Acid Labeling kit, Invitrogen (CA, USA), according to the manufacturer’s instructions. Five micrograms of CXCL4, or control molecules, were mixed with 10 µg of labeled DNA, in 15 µl of PBS and each suspension was put under a coverslip. Complex formation was visualized by confocal laser scanner microscopy (LSM). Acquisition of images was performed by a confocal microscope 510 Zeiss, objectives ×20, ×40, and ×60.

### Imaging of CXCL4–huDNA and CXCL4–bacDNA complexes in pDCs

To visualize the uptake of DNA into purified pDCs, we used Alexa Fluor 488-labeled huDNA or bacDNA. CXCL4 or control molecules were premixed with labeled huDNA or bacDNA for 10–15 min at room temperature. DNA complexes were given to the pDCs (10^5^ cells in 150 µl of culture medium) as well as the labeled DNA alone. After 1-h incubation in round-bottomed 96-well plates, pDCs were extensively washed and seeded again for a further 3 h. Fluorescence was directly analyzed by flow cytometry (see also Supplementary Fig. 9). In some experiments, pDCs were pretreated with a blocking anti-CXCR3 antibody at 10 µg ml^−1^ or isotype control antibody (both from R&D) or with human recombinant CXCL10 (10 µg ml^−1^) for 30 min, before adding the CXCL4–DNA or control complexes or DNA alone.

### Immunofluorescence and flow cytometry analysis

CXCR3 expression of purified pDCs was assessed by staining with a fluorochrome-conjugated anti-CXCR3 monoclonal antibody or isotype control (from B&D or eBiosciences). Cells were analyzed by flow cytometry (FACSCanto, Becton). Analysis was performed by FlowJo10.0.7 (Tristar, USA).

### Analysis of skin biopsies by LSM

Five-micrometer sections in paraffin of human SSc and HD skin biopsies were stained after de-paraffination in xylene (5 min, two times), followed by passages in absolute ethanol (3 min), 95% ethanol in water (3 min), 80% ethanol in water (3 min), 70% ethanol in water (3 min), and antigen retrival (5 min at 95 °C in 10 mM sodium citrate, pH 6.0). Slides were saturated with blocking buffer (PBS, 0.05% Tween20, 4% BSA) for 1 h at room temperature. The following antibodies were used: polyclonal rabbit anti-human CXCL4 (Abcam, ab9571^3^) or polyclonal mouse anti-human CXCL4 (Clone 170138 and control IgG2B, R&D), mouse or goat anti-human to CD303/BDCA2 from Miltenyi Biotech GmbH (Germany), or Novus Biological (DLEC/CLEC4C/BDCA-2), mouse anti-human neutrophil elastase (Millipore, clone AHN-10), polyclonal rabbit–anti-human myeloperoxidase (MPO) (Abcam, ab9535). Antibodies were added for 1 h at room temperature in a humidified chamber. Slides were washed three times with PBS 0.1% Tween 20 and incubated with the following secondary antibodies: donkey anti-rabbit IgG Alexa Fluor 488 or 568, goat or donkey anti-mouse Alexa Fluor 488 or 647, and donkey anti-goat Alexa Fluor 488 (Abcam). Appropriate isotype control or irrelevant polyclonal antibodies were also used. After washing, slides were mounted in Prolong Gold antifade media containing a DNA dye (DAPI, Molecular Probes) before analysis with a confocal microscope, objectives ×20, or ×40 and ×60 in oil immersion (FV1000 Olympus, Tokyo, Japan), by Olympus planapo objective ×40 or ×60 oil A.N. 1.42. Excitation lights: LaserDapi 408 nm for DAPI, Argon Ion Laser (488 nm) for Alexa 488, Diode Laser HeNe (561 nm) for Alexa 568, and Red Diode Laser (638 nm) for Alexa 647. DAPI emission was recorded from 415 to 485 nm, Alexa 488 emission from 495 to 550 nm, Alexa 568 from 583 to 628 nm, and Alexa 647 from 634 to 750 nm. Some images were acquired with a Leica TCS SP2 apparatus. Images recorded had an optical thickness of 0.3 µm.

### SAXS methods

For SAXS experiments, *E. coli* DNA was ethanol precipitated and resuspended in physiological buffer (140 mM NaCl + 10 mM HEPES, pH 7.4) to 5 mg ml^−1^. HuDNA was used directly unfragmented or fragmented by sonication (as described above). Self-assembled protein–DNA complexes were formed by incubating the CXCL4 or LL37 with DNA at isoelectric peptide-to-DNA charge ratios (P/DNA = 1/1) in microcentrifuge tubes. Complexes were vortexed at low speeds (900 RPM) for 1 h or until strong precipitates formed. After thorough mixing and centrifugation, precipitated complexes are transferred to 1.5-mm quartz capillaries (Hilgenberg GmbH, Mark-tubes) and hermetically sealed using an oxygen torch. The structures of CXCL4–DNA and the related complexes were solved using SAXS. Experiments were performed at the Stanford Synchrotron Radiation Lightsource (SSRL, Beamline 4-2) using monochromatic X-rays with an energy of 9 keV. A Rayonix MX225-HE detector (pixel size 73.2 μm) was used to measure the scattered radiation. Independent identical samples were prepared and measured over multiple separate experiments (*n* ≥ 3). 2D powder diffraction patterns were azimuthally integrated using the Nika 1.76 package for Igor Pro 7.04 and FIT2D^65,66^. SAXS data were analyzed by plotting scattering intensity *I*(*q*) against the momentum transfer *q* in Mathematica.

Structures of the CXCL4–DNA complexes were solved by measuring the *q*-positions of all peaks and comparing them with the permitted reflections for phases with varying symmetries. Peak positions were accurately measured by fitting diffraction peaks to a squared Lorentzian. SAXS patterns with a single major peak at position *q*_1_ corresponds to a liquid-crystalline DNA structure with short-range order. The lattice parameter *d* indicates the inter-DNA spacing between parallel DNA columns and is calculated from the first peak position by the formula *q*_1_ = 2*π*/*d*. LL37–DNA complexes are more ordered. For square columnar DNA lattices from LL37–DNA nanocrystalline complexes, we observe peaks at $q_{hk} = \frac{{2\pi }}{d}\sqrt {h^2 + k^2}$ where (*h*,*k*) are the Miller indices and *d* is the lattice parameter corresponding to the inter-DNA spacing. Typical square lattices will have reflections at and with a ratio of 1:√2. For hexagonal columnar DNA lattices, we observe peaks at $q_{hk} = \frac{{2\pi }}{d}\sqrt {\frac{4}{3}(h^2 + k^2 + hk)}$. Typical hexagonal lattices will have reflections at *q*_10_, *q*_11_, and *q*_20_ with ratios of 1:√3:2. The lattice parameters were calculated by linear regression through points corresponding to measured and theoretical peaks. Procedures to assign these structural phases are similar to those found here^66–68^.

To calculate the domain size of the nanocrystalline complexes, the first peak was fitted to a squared Lorentzian using least-squares regression in Mathematica (*q*_1_ is the location of the first peak and *h* is the peak width)^28^

$$S\left( q \right) = \frac{{h^3}}{{4\pi \left( {\left| {q - q_1} \right|^2 + \left( {\frac{h}{2}} \right)^2} \right)^2}}$$

We extract the peak width *h* and calculate the average domain size *L* using Warren’s approximation^67^. The domain size is related to *h* via the following equation^68^:

$$L(h) = \left( {8\pi } \right)^{1/2}/(h/2)$$

For liquid-crystalline structures with short-ranged order, we need to use a different expression for the estimated domain size. The occurrence of a single broad diffraction feature in *S*(*q*) suggests a disordered system with a short-ranged exponential decay of positional correlations. Here, *S*(*q*) has a Lorentzian form. After powder averaging over all solid angles, we approximate the domain size as

$$L = \frac{{2^{1/2}}}{{h/2}}$$

The program used for analyzing the synchrotron X-ray data is FIT2D. It is freely available and downloadable from http://www.esrf.eu/computing/scientific/FIT2D/.

### Statistical analysis

Differences between mean values were assessed by Student’s *t* test for unpaired samples or Mann–Whitney to compare SSc, SLE patients, and HD sera/plasma for content of IFN-α or CXCL4 or control molecules (one-tailed or two-tailed). Student’s *t* test for paired samples or Wilcoxon signed-rank test were used to compare the effects of CXCL4 alone or in complex with DNA or SSc plasma, plus/without DNA, and control molecules/plasma, on purified pDCs. Pearson’s correlation coefficient was used to assess the correlation between IFN-α levels and levels of CXCL4, S100A8, S100A9, LL37, or Scl70 (antitopoisomerase autoantibodies) in SSc or control individuals. Statistical significance was set at *P* < 0.05.

### Reporting summary

Further information on experimental design is available in the Nature Research Reporting Summary linked to this article.

## Supplementary information

Supplementary Information

Reporting Summary

## Data Availability

Data supporting the findings of this study are available within the article and its Supplementary Information files and from the corresponding authors upon reasonable request.

## Code availability

Code for data analysis will be provided upon reasonable request.
