# Supplementary material for: CXCL4 assembles DNA into liquid crystalline complexes to amplify TLR9-mediated interferon-α production in systemic sclerosis
Source: Nat Commun. 2019 May 1;10:1731. doi: 10.1038/s41467-019-09683-z (PMC6494823; doi:10.1038/s41467-019-09683-z)
Supplement: Supplementary file 1 — Supplementary Information [file 41467_2019_9683_MOESM1_ESM.pdf]

CXCL4 assembles DNA into liquid crystalline complexes to amplify TLR9-mediated interferon-α production in systemic sclerosis

Lande R et al.

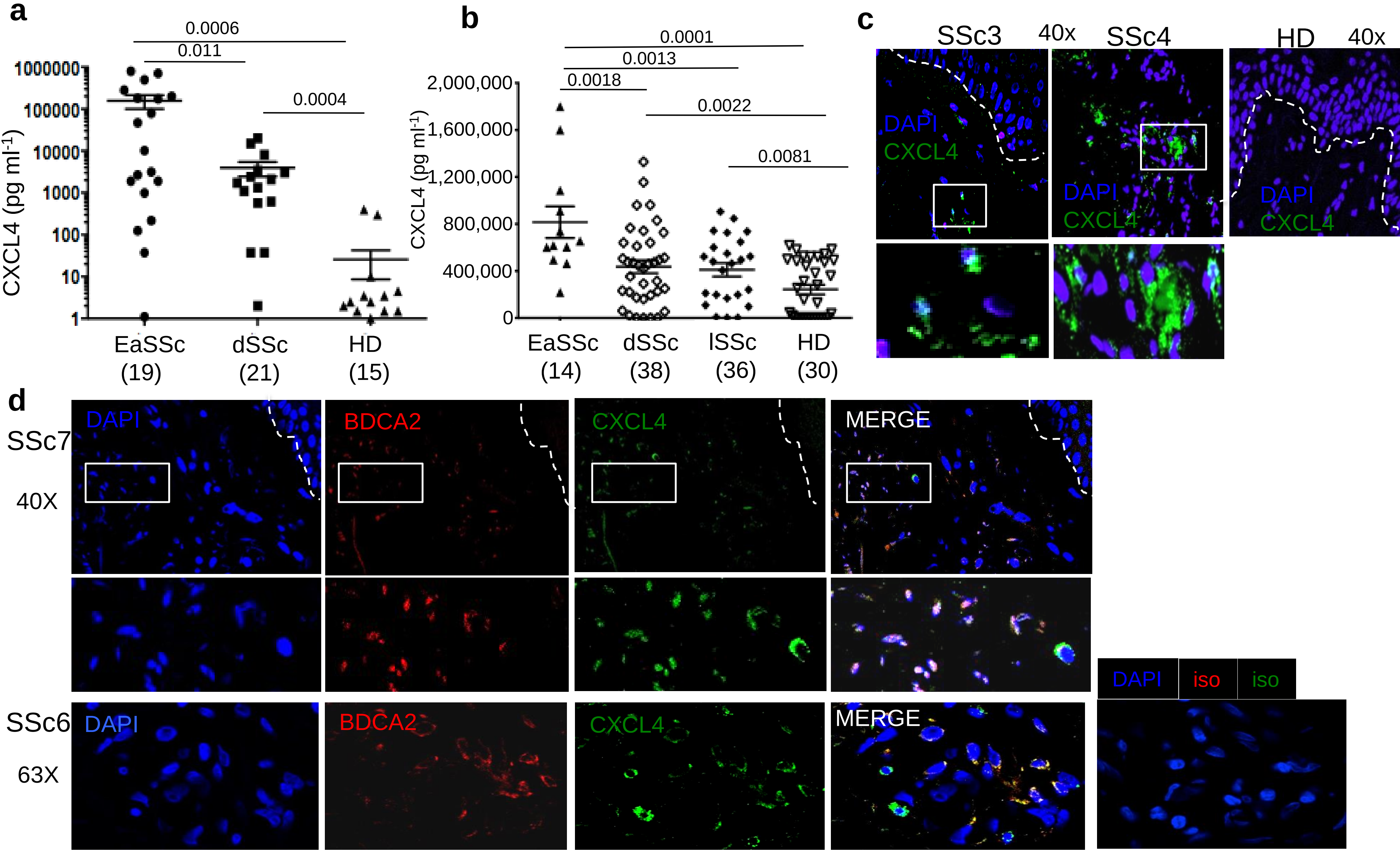

Supplementary Figure 1. CXCL4 is up-regulated in blood and skin of SSc and not in HD. CXCL4 levels in SSc plasma **(a)** and sera **(b)** of SSc patients stratified on the bases of SSc forms as in Figure 1. Horizontal bars represent the mean, vertical bars SEM, P values by Student's t-test for unpaired sample (two-tailed). **(c)** Confocal images of staining of CXCL4 in the skin of SSc patients and in HD; representative confocal images of stainings of 22 skin biopsies from SSc and 11 from HD. **(d)** Co-localization of CXCL4 with pDCs (BDCA2) in the SSc skin; representative confocal images of staining of two SSc biopsies of ten performed.

Supplementary Table 1: Clinical characteristics of the studied patients' cohorts

a) Blood	SSc (38)	SSc (88)(Repl. cohort)	SLE (85)	Controls (60)
Age, mean (range) years	50.9 (27–77)	58.7 (28-74)	48 (25–70)	50 (26–60)
Sex (M/F)	6/74	6/74	2/18	5/35
Disease duration, mean (range) months	120.7 (1–456)	100.2 (1-550)	48 (12–500)	N/A
SLEDAI	-	-	9.5 (0-21)	N/A
Form (limited/diffuse)	3/28	53/27	N/A	N/A
MRSS (mean, range)	1.0 (0.5-4.5)	3 (0-5)	N/A	N/A
ANA positivity (yes/no)	34/4	84/4	18/2	N/A
ANA specificity (ATA/ACA)	28/6	60/24	N/A	N/A
DLCO, mean (range), % of Reference	77 (68–85)	78 (71-89)	N/A	N/A
Synovitis	23%	30%	N/A	N/A
Myositis (CK elevation)	1%	30%	N/A	N/A
DU	20%	57%	N/A	N/A
Tendon friction rubs	28%	40%	N/A	N/A
ILD	50%	50%	N/A	N/A
GORD	50%	59%	N/A	N/A
Prednisolone use (%)	60%	75%	59%	None
Current use of IS (n)	5	15	5	None
Previous use of IS (n) (≥6 weeks before sampling)	8	30	5	None

b) Skin biopsies	SSc (23)	Controls (11)
Age, mean (range) years	51.9 (30–80)	50.5 (27-70)
Sex (M/F)	3/20	3/8
Disease duration, mean (range) months	55.0 (6–550)	N/A
Form (limited/diffuse)	7/16	N/A
EaSSc/long lasting	6/17	N/A
MRSS (mean, range)	18.4 (0–34)	N/A
ANA positivity (yes/no)	21/2	N/A
ANA specificity (ATA/ACA)	15/3	N/A
DLCO, mean (range), % of Reference	N/A	N/A
DU	85%	N/A
Tendon friction rubs	40%	N/A
ILD	40%	N/A
GORD	25%	N/A

DU and synovitis were clinically defined, disease duration refers to the time from the onset of the first non-Raynaud's disease manifestation, ILD was assessed by high resolution CT scan, GORD was determined by gastroscopy.
ACA, anti-centromere antibody; ANA, anti-nuclear antibodies; ATA, anti-topoisomerase, antibody; CK, creatine kinase; DLCO, diffusing capacity of the lungs for carbon, monoxide; DU, digital ulcer; GORD, gastro-oesophageal reflux disease; ILD, interstitial, lung disease; IS, immunosuppressant agents; MRSS, modified Rodnan skin score; N/A, not available; SSc, systemic sclerosis.

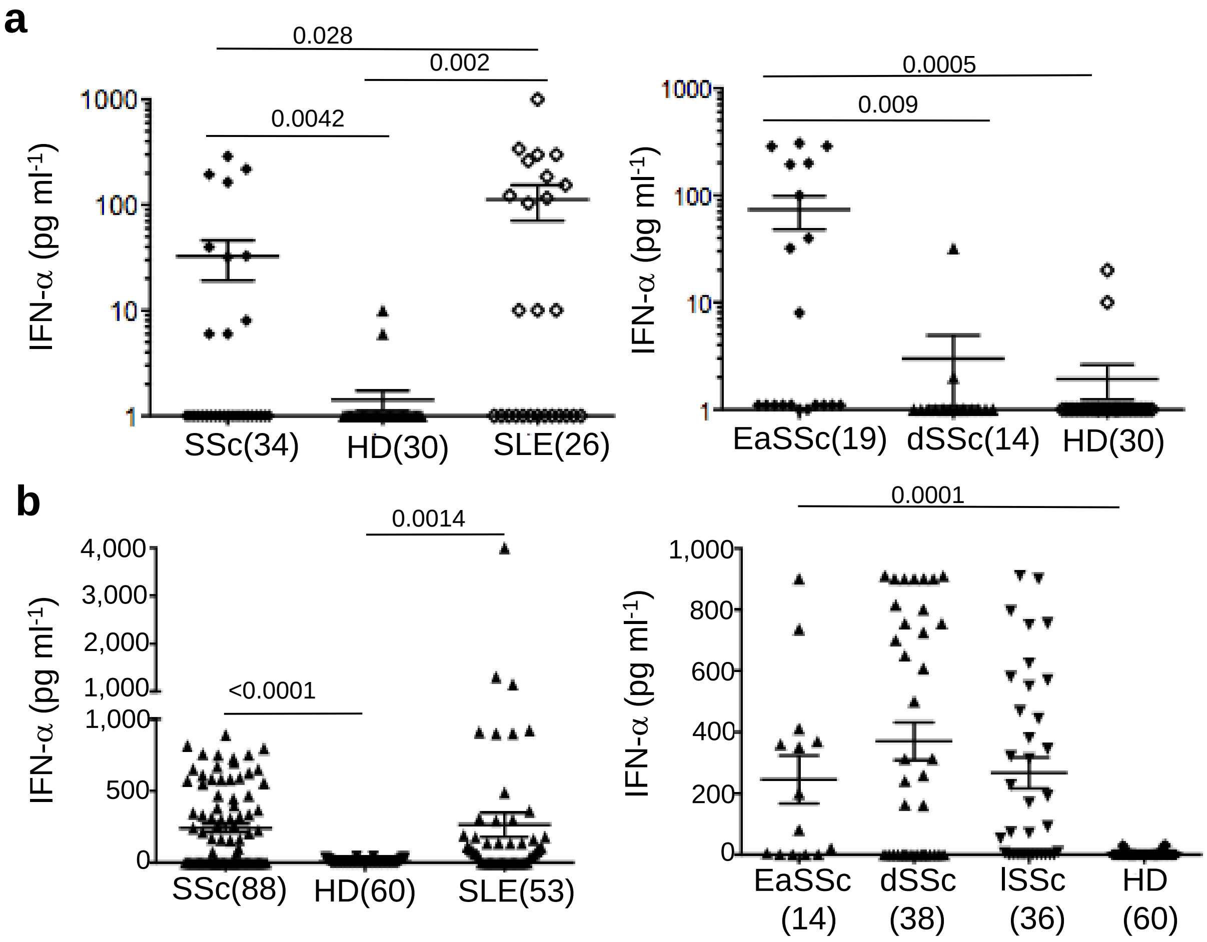

Supplementary Figure 2. Expression of IFN-α and correlation with CXCL4 in SSc plasma/sera. SSc, HD and SLE plasma **(a)** and sera **(b)** were diluted 1:4 and tested for levels of IFN-α by ELISA (in the right panels, IFN-α expression by SSc subtypes as in Fig. 1). Ten out 34 (29%) **(a)**, 48 out of 88 SSc patients (54%) **(b)**, 13 out of 26 (50%) **(a)** and 32 of 53 SLE patients (60%) **(b)**, showed an IFN-α-signature. Horizontal bars represent the mean, vertical bars are SEM, P values by Student's t-test for unpaired samples (two-tailed), number of individuals tested indicated.

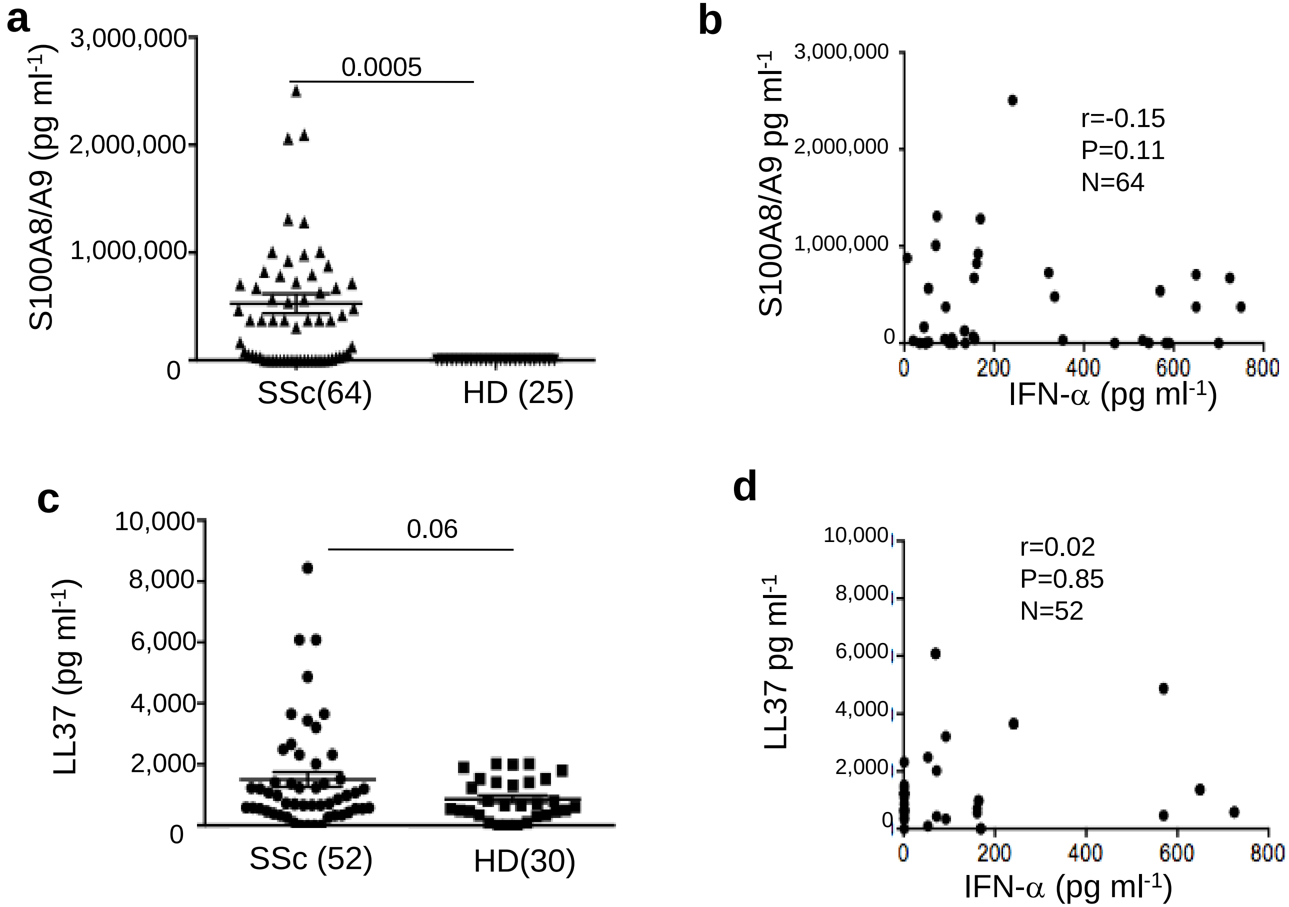

Supplementary Figure 3. S100A8/9 or LL37 levels did not correlate with IFN-α levels in SSc. SSc expression levels of S100/A8/9 **(a)** or LL37 **(c)** in SSc patients and HD measured buy ELISA test. S100A8/9 (**b**), and LL37, **(d)** were correlated to levels of IFN-α in SSc by Pearson's correlation analysis. Coefficient of correlation "r" are indicated, as well as significance P and sample size N.

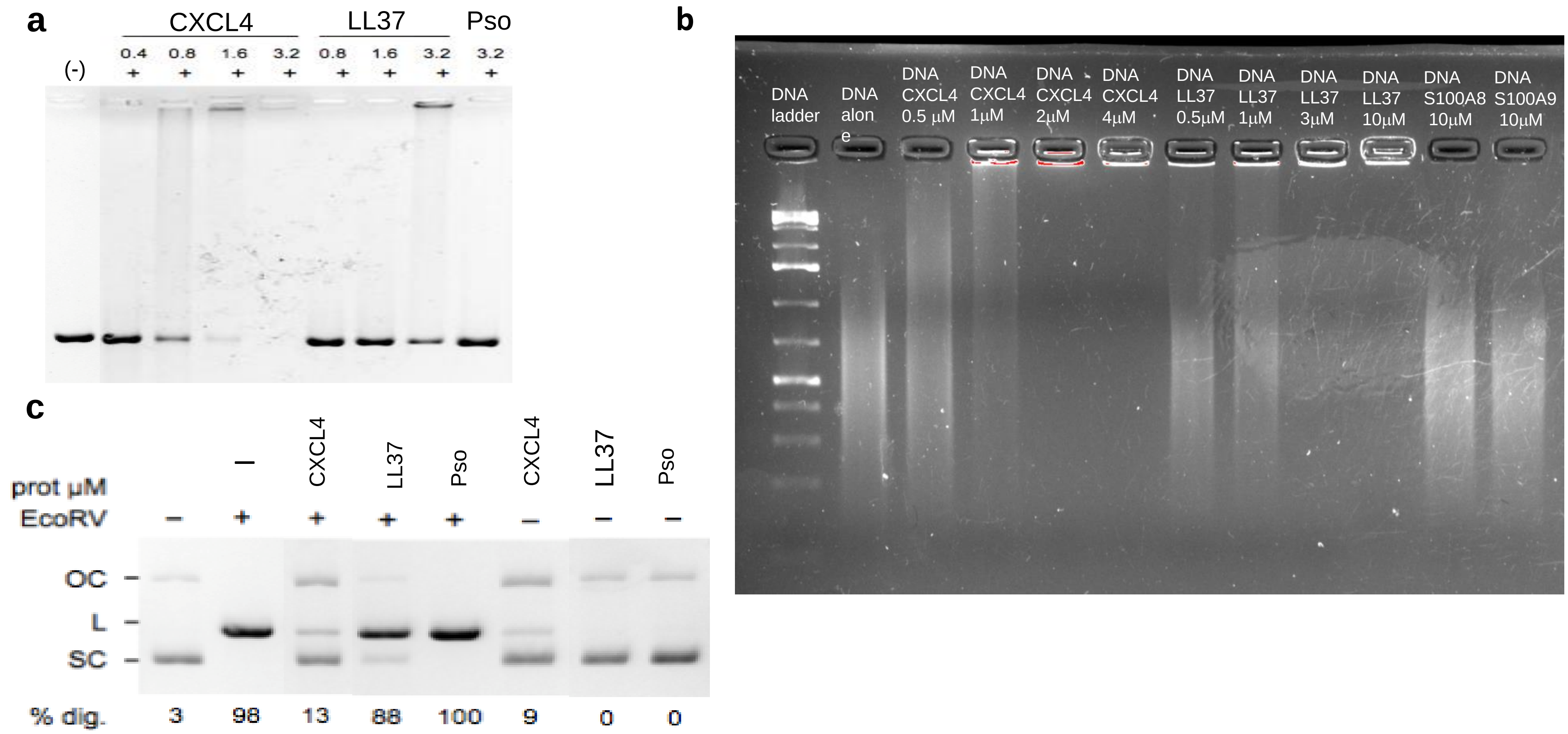

Supplementary Figure 4. CXCL4 binds DNA and protects DNA from enzymatic degradation. (a) CXCL4 or LL37 or psoriasin (Pso) at the indicated concentrations (µM) were mixed with linear pDB29 DNA in DPBS supplemented with 0.5mM $MgCl_2$ and 0.9mM $CaCl_2$ and incubated 30 minutes at 37°C. The mixtures were run on a 0.8% agarose gel and stained with ethidium bromide to visualize binding of the peptide to DNA as delayed DNA migration. One representative experiment is shown out of three**. (b)** Delayed human DNA migration in the presence of different doses of CXCL4 or LL37, visualized on a 2% agarose gel. One representative experiment of four performed. (**c**) 15 ng (=1.97 nM) of circular pDB29 DNA was incubated for 30 minutes at 37°C with 3µM CXCL4, LL37, or psoriasin (Pso) in the presence or absence of 0.55 U of EcoRV (0.4µM = 1 AMP molecule per 28.3 bp of the plasmid DNA). Cleavage of pDB29 DNA is visualized by DNA after extraction and precipitation, by separation on a 1% agarose gel. Percent of digested DNA (% dig.), determined by densitometry analysis, is indicated. The control migration positions of relaxed-circular (OC), supercoiled-circular (SC) and linear DNA (L) are indicated on the left. Results are representative of two independent experiments.

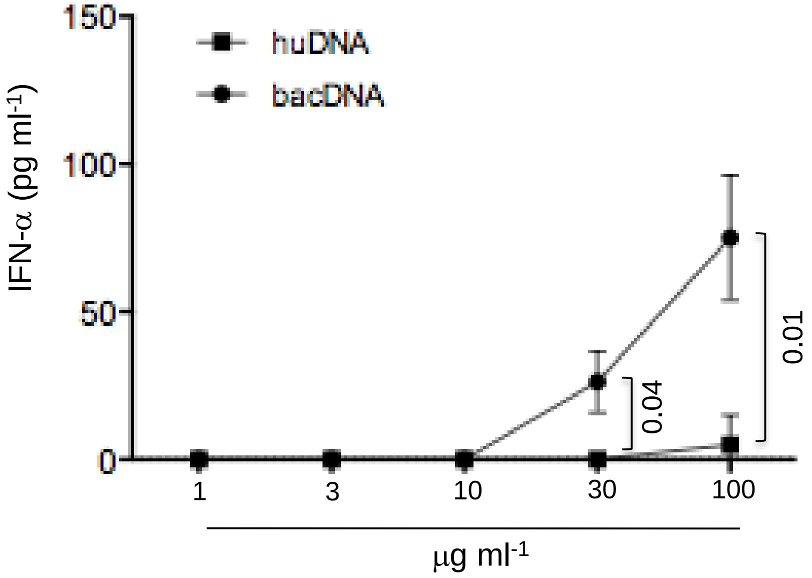

Supplementary Figure 5. Natural DNA alone is poorly stimulatory for pDCs. Dose-response curve of IFN-α production by pDCs stimulated with increasing concentrations of huDNA and bacDNA in the absence of CXCL4 or any other peptide. Significant activation of pDCs was observed at 30 μg ml^{-1} ($P = 0.04$, $N=7$) and 100 μg ml^{-1} ($P = 0.01$, $N=7$) concentrations of bacDNA, but not at concentrations ≤10 μg ml^{-1}. IFN-α production values measured in untreated pDCs = 0. P values by student's t-test for unpaired samples (two-tailed).

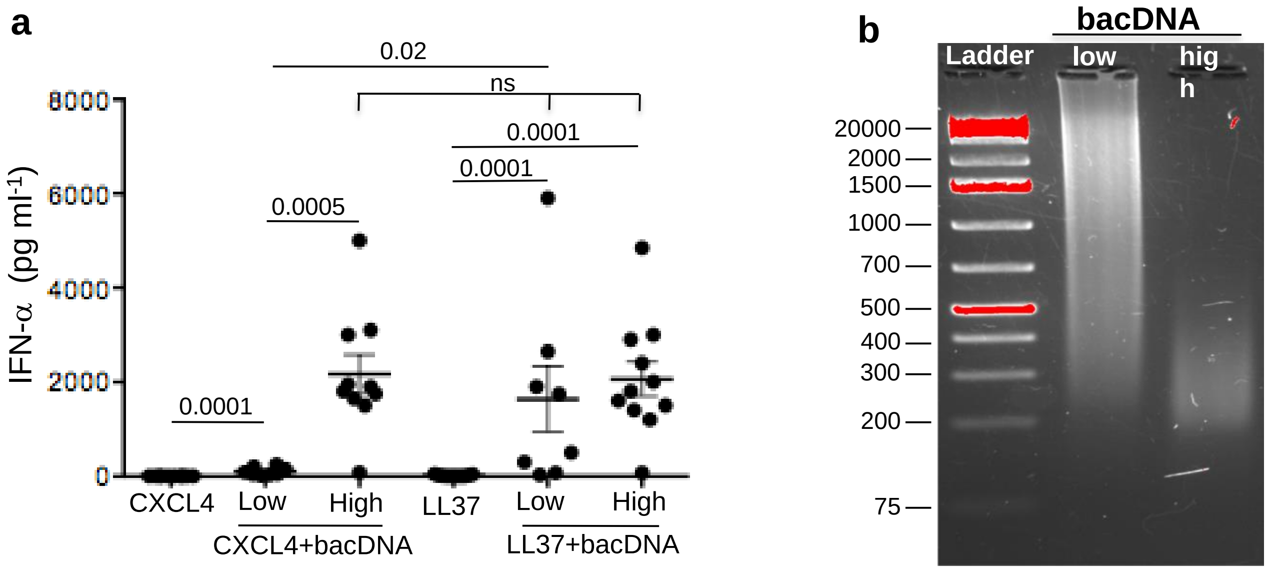

Supplementary Figure 6. Stimulation of pDCs by CXCL4-bacDNA complexes is also sensitive to the degree of DNA fragmentation. (a) PDCs were cultured with either low or high fragmented *E. coli* DNA (bacDNA) preparations in complex or not, with CXCL4 (1 µM) or LL37 (10 µM) and IFN-α production was analyzed as in Figure 3. Horizontal bars represent the mean, vertical bars are SEM, P values from Student's t-test for paired samples (two-tailed); **(b)** 2% agarose gel shows the degree of fragmentation of the two *E.Coli* DNA preparations used in functional assays of panel (a). Fragment sizes of the ladder (in base pairs) are indicated on the lelf side of the gel.

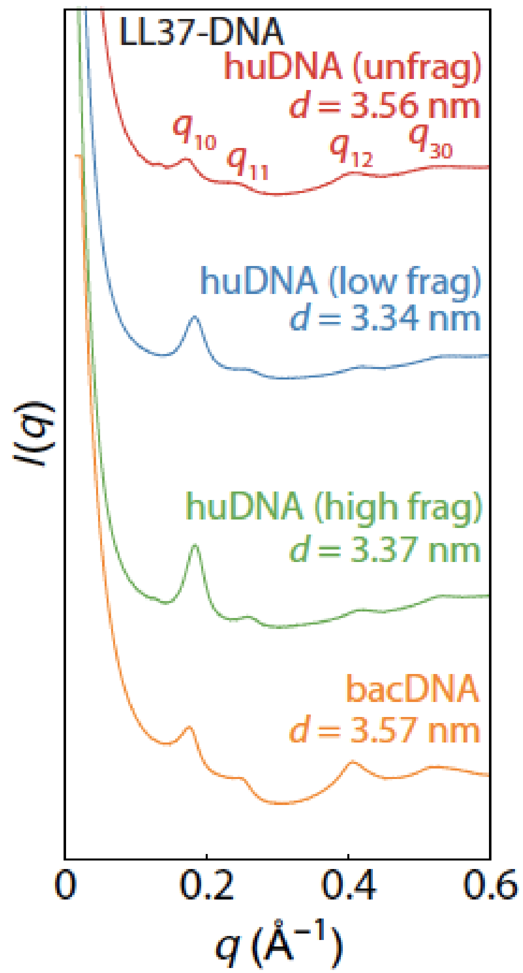

Supplementary Figure 7. LL37 organizes DNA into square columnar lattices to amplify TLR9 activation. SAXS data of LL37 bound to huDNA and bacDNA (as in Figure 6d): First peak position q_1 and inter-DNA spacings (*d*) are indicated.

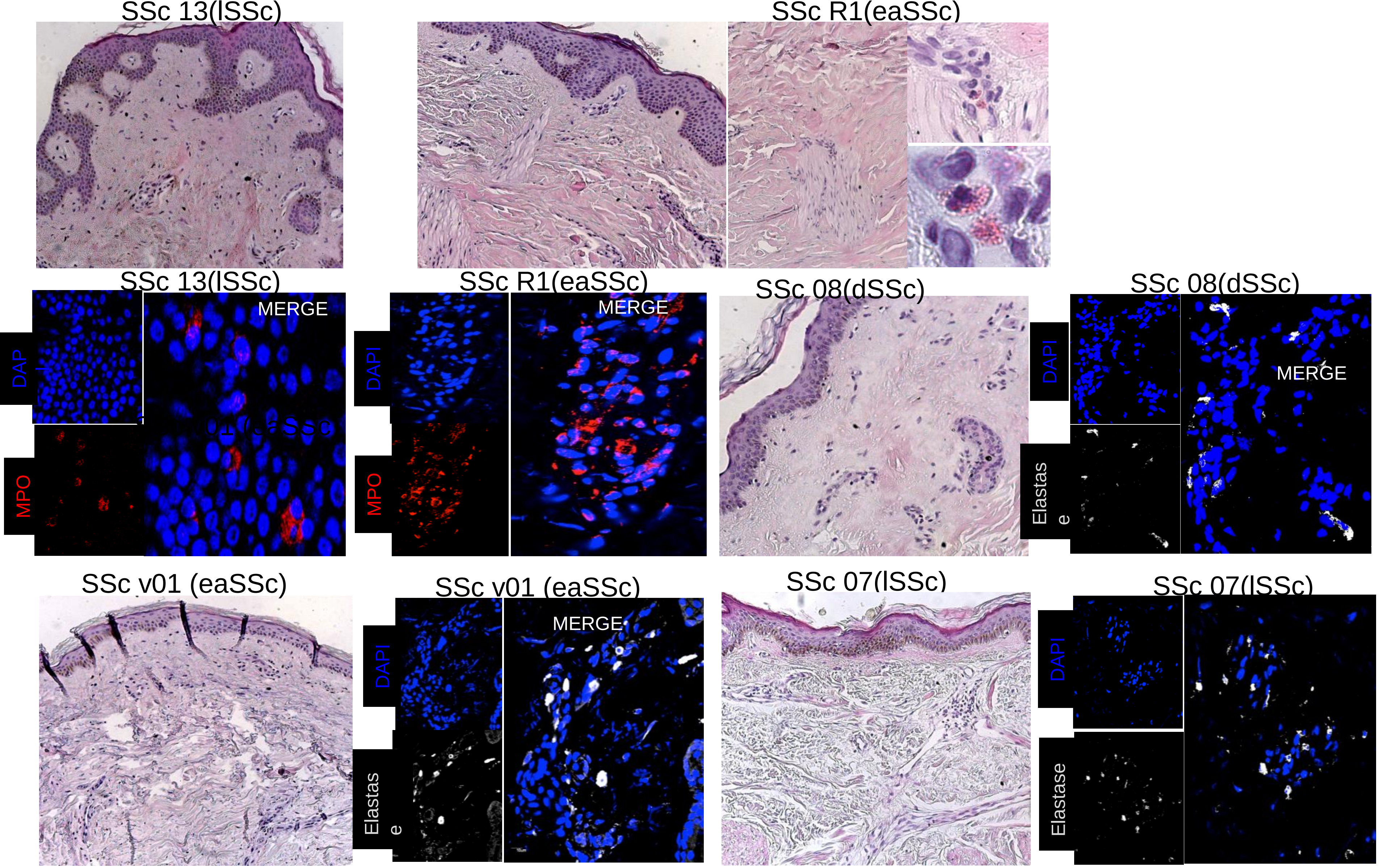

Supplementary Figure 8. Neutrophils are represented in skin infiltrate of SSc patients. Confocal images of DNA (blue) and neutrophil elastase (grey) or MPO (red) in SSc skin, and H&E staining (original magnification: 10x) of the correspondent SSc skin biopsies. Five representative SSc skin biopsies of ten analyzed are shown as confocal images (original magnification: 63x).

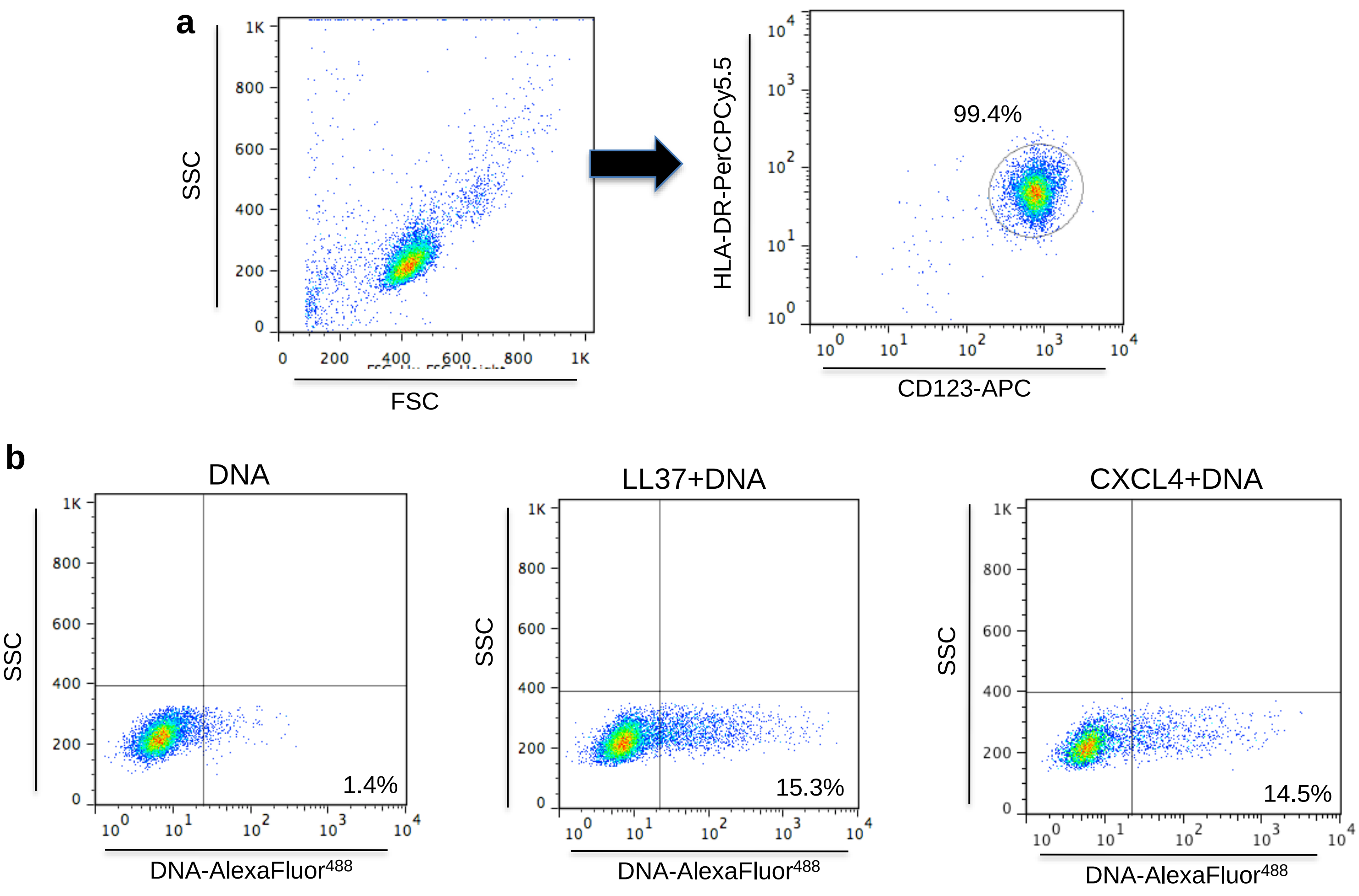

Supplementary Figure 9. Representative gating scheme of plasmacytoid dendritic cell purification and DNA uptake. (a) Flow cytometry data of PDCs purified from blood as described in Methods and stained with the indicated fluorochrome-conjugated antibodies to assess the percentage of cell purity. **(b)** PDCs purified in panel a were cultured in vitro with fluorochrome-conjugated DNA alone (DNA) or DNA complexed with LL37 (LL37+DNA) or CXCL4 (CXCL4+DNA). DNA uptake was determined by flow cytometry after 3h. The side scatter parameter is indicated as SSC on y-axis. One representative experiment out of five is shown.
