# Supplementary material for: CXCL4 assembles DNA into liquid crystalline complexes to amplify TLR9-mediated interferon-α production in systemic sclerosis
Source: Nat Commun. 2019 May 1;10:1731. doi: 10.1038/s41467-019-09683-z (PMC6494823; doi:10.1038/s41467-019-09683-z)
Supplement: Supplementary file 2 — Reporting Summary [file 41467_2019_9683_MOESM2_ESM.pdf]

nature research

Corresponding author(s): Loredana Frasca, Roberto Lande, Gerard CL Wong

Last updated by author(s): 03.11.2019

Reporting Summary

Nature Research wishes to improve the reproducibility of the work that we publish. This form provides structure for consistency and transparency in reporting. For further information on Nature Research policies, see Authors & Referees and the Editorial Policy Checklist.

Statistics

For all statistical analyses, confirm that the following items are present in the figure legend, table legend, main text, or Methods section.

n/a	Confirmed	
☐	☒	The exact sample size (*n*) for each experimental group/condition, given as a discrete number and unit of measurement
☐	☒	A statement on whether measurements were taken from distinct samples or whether the same sample was measured repeatedly
☐	☒	The statistical test(s) used AND whether they are one- or two-sided<br>*Only common tests should be described solely by name; describe more complex techniques in the Methods section.*
☒	☐	A description of all covariates tested
☒	☐	A description of any assumptions or corrections, such as tests of normality and adjustment for multiple comparisons
☒	☐	A full description of the statistical parameters including central tendency (e.g. means) or other basic estimates (e.g. regression coefficient) AND variation (e.g. standard deviation) or associated estimates of uncertainty (e.g. confidence intervals)
☐	☒	For null hypothesis testing, the test statistic (e.g. *F*, *t*, *r*) with confidence intervals, effect sizes, degrees of freedom and *P* value noted<br>*Give P values as exact values whenever suitable.*
☒	☐	For Bayesian analysis, information on the choice of priors and Markov chain Monte Carlo settings
☒	☐	For hierarchical and complex designs, identification of the appropriate level for tests and full reporting of outcomes
☐	☒	Estimates of effect sizes (e.g. Cohen's *d*, Pearson's *r*), indicating how they were calculated

Our web collection on statistics for biologists contains articles on many of the points above.

Software and code

Policy information about availability of computer code

Data collection	Images were taken with the built-in softwares of Leica TCS SP2, FV1000 Olympus, Zeiss 510 confocal microscopes as described in Methods. Flow cytometry data were collected with a BD FACSDiva sofware. SAXS experiments were performed at the Stanford Synchrotron Radiation Lightsource (SSRL. Beamline 4-2).
Data analysis	Confocal images were analysed by a Leica software 2.6 and by ZEISS 510 software. Flow cytometry data were analysed by FlowJo 7.6.5. X-ray data was analysed using Igor Pro 7 and Mathematica 11 as detailed in Methods.

For manuscripts utilizing custom algorithms or software that are central to the research but not yet described in published literature, software must be made available to editors/reviewers. We strongly encourage code deposition in a community repository (e.g. GitHub). See the Nature Research guidelines for submitting code & software for further information.

Data

Policy information about availability of data

All manuscripts must include a data availability statement. This statement should provide the following information, where applicable:

- Accession codes, unique identifiers, or web links for publicly available datasets
- A list of figures that have associated raw data
- A description of any restrictions on data availability

All data generated in this study are available within the Article and in the Supplementary Information files. Additional data are available from the corresponding authors upon request.

Field-specific reporting

Please select the one below that is the best fit for your research. If you are not sure, read the appropriate sections before making your selection.

☒ Life sciences ☐ Behavioural & social sciences ☐ Ecological, evolutionary & environmental sciences

For a reference copy of the document with all sections, see nature.com/documents/nr-reporting-summary-flat.pdf

Life sciences study design

All studies must disclose on these points even when the disclosure is negative.

Sample size	We did not use any statistical method to predetermine sample size for patients because the patients' cohorts were composed of previously enrolled subjects by the clinicians. Sample sizes are similar to those used in previous published clinical studies.
Data exclusions	No data were excluded
Replication	About SAXS data, Independent identical samples were prepared and measured over multiple separate experiments ($n \geq 3$) as detailed in Methods. CXCL4 levels, and related correlations, were measured by ELISA in the plasma of SSc (Discovery cohort) and replicated in the sera of a second cohort of SSc patients (Replication cohort) as specified in the figure legends. In vitro experiments were confirmed by multiple replicates as detailed in the figure legends.
Randomization	N/A
Blinding	All experiments of this study generated a direct measurement of the levels analysed. Then, the analysis by blinded investigators was not relevant.

Reporting for specific materials, systems and methods

We require information from authors about some types of materials, experimental systems and methods used in many studies. Here, indicate whether each material, system or method listed is relevant to your study. If you are not sure if a list item applies to your research, read the appropriate section before selecting a response.

Materials & experimental systems

n/a	Involved in the study	
☐	☒	Antibodies
☒	☐	Eukaryotic cell lines
☒	☐	Palaeontology
☒	☐	Animals and other organisms
☐	☒	Human research participants
☒	☐	Clinical data

Methods

n/a	Involved in the study	
☒	☐	ChIP-seq
☐	☒	Flow cytometry
☒	☐	MRI-based neuroimaging

Antibodies

Antibodies used	anti-CD32 (clone AT10, Abcam); anti-CXCR3 PE (clone 1C6, BD); anti-Mx1 (clone OTI2G12, Novus Biologicals); anti-CXCL4 (rabbit polyclonal IgG, ab9561, Abcam); anti-CXCR3 (inhibitory, clone 49801, R&D); anti-CXCL4 (inhibitory, ab9561, Abcam); anti-BDCA2 (AF1376, R&D); anti-S100A8/MRP8 (ABF125, Merck Millipore). Isotype controls: mouse IgG1 PE (556650, BD); goat polyclonal isotype control (AB-108-C, R&D); mouse IgG isotype control (NBP1-97019); mouse IgG1 isotype control (ab185801, Abcam); rabbit IgG polyclonal isotype control (ab37415, Abcam); mouse IgG1 isotype control (clone 11711, R&D).
Validation	anti-S100A8/MRP8 (ABF125, Merck Millipore) was validated for use in WB, IHC and Activity Assay by the manufacturer.<br>anti-CXCR3 clone 49801 (R&D) was validated for neutralization activity by the manufacturer.<br>anti-CXCL4 rabbit polyclonal (ab9561, Abcam) is validated by the manufacturer for WB, ELISA, inhibition of the biological activity of PF4, immunostaining on paraffinated tissue sections.<br>Anti-CD32 (clone AT10, Abcam) is validated for blocking and neutralising assay by the manufacturer.<br>Fluorochrome-conjugated anti-CXCR3 (clone 1C6, BD Biosciences) is validated for flow cytometry.<br>Anti-BDCA2 (AF1376, R&D) is validated for WB, flow cytometry and immunostaining on tissues.<br>Anti-Mx1 (clone OTI2G12, Novus Biologicals) is validated for WB, immunofluorescence and immunoistochemistry

Human research participants

Policy information about studies involving human research participants

Population characteristics	For this study we used a SSc cohort, a SSc replication cohort, a SLE cohort and a group of healty donors (control). The clinic

Population characteristics	characteristics of these subjects are detailed in Supplementary Table 1. Patients with SSc satisfied the ACR/EULAR 2013 classification criteria for SSc. This study was approved by the ethical committee of the institutions involved ("Commission cantonale d'éthique de la recherche", Geneva, Switzerland; the Ethics Committee of the Sapienza University, Rome, and University Hospital of Bordeaux, France) and was conducted according to the Declaration of Helsinki. Informed, written consent was obtained from all participants according to the declaration of Helsinki.
Recruitment	N/A
Ethics oversight	This study was approved by the ethical committee of the institutions involved ("Commission cantonale d'éthique de la recherche", Geneva, Switzerland; the Ethics Committee of the Sapienza University, Rome, and University Hospital of Bordeaux, France) and was conducted according to the Declaration of Helsinki. Informed, written consent was obtained from all participants according to the declaration of Helsinki.

Note that full information on the approval of the study protocol must also be provided in the manuscript.

Flow Cytometry

Plots

Confirm that:

- [x] The axis labels state the marker and fluorochrome used (e.g. CD4-FITC).
- [x] The axis scales are clearly visible. Include numbers along axes only for bottom left plot of group (a 'group' is an analysis of identical markers).
- [ ] All plots are contour plots with outliers or pseudocolor plots.
- [x] A numerical value for number of cells or percentage (with statistics) is provided.

Methodology

Sample preparation	- Buffy coat from healthy donors were provided by the Blood center in Lausanne (CH) and Rome (IT). Plasmacytoid cells (PDC) were purified from buffy coat by using Diamond Plasmacytoid Dendritic Cell Isolation II Kit (Miltenyi Biotec) to obtain 99% of purity.<br>- Five-µm sections in paraffin of human SSc and HD skin biopsies were stained after de-paraffination in xilene (5 minutes, two times), followed by passages in: absolute ethanol (3 minutes), 95% ethanol in water (3 minutes), 80% ethanol in water (3 minutes), 70% ethanol in water (3 minutes), and antigen retrival (5 minutes at 95°C in 10 mM sodium citrate, pH 6.0). Slides were saturated with blocking buffer (PBS, 0.05% tween20, 4% BSA) for 1 hour at room temperature, followed by antibody staining.<br>- For SAXS experiments, E. coli DNA was ethanol precipitated and resuspended in physiological buffer (140 mM NaCl + 10 mM HEPES, pH 7.4) to 5 mg/ml. HuDNA was used directly unfragmented or fragmented by sonication. Self-assembled protein- DNA complexes were formed by incubating the CXCL4 or LL37 with DNA at isoelectric peptideto-DNA charge ratios (P/DNA = 1/1) in microcentrifuge tubes. Complexes were vortexed at low speeds (900 RPM) for 1 hour or until strong precipitates formed. After thorough mixing and centrifugation, precipitated complexes are transferred to 1.5 mm quartz capillaries (Hilgenberg GmbH, Mark-tubes) and hermetically sealed using an oxygen torch. The structures of CXCL4-DNA and related complexes were solved using SAXS.
Instrument	Flow cytometry data were collected by FACSCanto (BD).<br>For confocal microscopy images a FV1000 Olympus (Tokyo, Japan) and a Leica TCS SP2 apparatus were used.<br>For SAXS experiments we used the Stanford Synchrotron Radiation Lightsource (SSRL, Beamline 4-2) using monochromatic X-rays with an energy of 9 keV. A Rayonix MX225-HE detector (pixel size 73.2 µm) was used to measure the scattered radiation
Software	Flow cytometry data were acquired by a FACSDiva software (BD), and analyzed by FlowJo (version 10.0.7, BD)
Cell population abundance	Plasmacytoid cells were purified from buffy coat by using Diamond Plasmacytoid Dendritic Cell Isolation Kit (Miltenyi Biotec) to obtain 99% pure pDCs. Purity was evaluated by staining with fluorochrome-conjugated anti-CD123 and anti-BDCA2 antibodies.
Gating strategy	All cells purified by Diamond Plasmacytoid Dendritic Cell Isolation Kit were plasmacytoid dendritic cells as previously described.

- [ ] Tick this box to confirm that a figure exemplifying the gating strategy is provided in the Supplementary Information.
